# Supplementary material for: Craniofacial ontogeny in Tylosaurinae
Source: PeerJ. 2020 Oct 20;8:e10145. doi: 10.7717/peerj.10145 (PMC7583613; doi:10.7717/peerj.10145)
Supplement: Supplemental Information 3 — Descriptions and exemplar specimens are included for each state. The characters are numbered as they appear in the data matrix and are organized by skull region. Character states were kept consistent across taxa. Clarifications about measurements, sources, etc. are given in a note below the character descriptions. [file peerj-08-10145-s003.docx]

**General Skull Characters (1 character)**

**1. Total Skull Length (TSL):** less than 400 mm (0), 400–799 mm (1), 800–999 mm (2), 1000–1399 mm (3), greater than or equal to 1400 mm (4).

**Exemplars:** FHSM VP-78 (0), FHSM VP-2295 (1), FGM V-43 (2), FHSM VP-3 (3),

KUVP 5033 (4).

**Note:** Total skull length was measured from the tip of the premaxillary rostrum to the posterior edge of the occipital condyle.

**Premaxilla (6 characters)**

**2. Premaxilla, predental rostrum, presence:** absent (0), present (1).

**Exemplars:** FHSM VP-3 (1). No specimen in this project exhibits state 0.

**Note:** This is a phylogenetic character from Bell (1997; character number 1); state 1 is diagnostic for *Tylosaurus*.

**3. Premaxilla, predental rostrum, predental rostrum length to TSL:** less than 5% TSL, greater than or equal to 5% TSL.

**Exemplars:** FHSM VP-2295 (0), FHSM VP-3 (1).

**Note:** Predental rostrum length was measured as the length from the tip of the premaxilla to the base of the first premaxillary tooth.

**4. Premaxilla, predental rostrum shape, lateral and dorsal views:** acute (0); rounded, distinct knob present (1).

**Exemplars:** AMNH FARB 4909 (0), FMNH P15144 (1).

**Note:** This character is figured in Figure S2. It was identified as ontogenetically variable in *Mosasaurus hoffmannii* by Mulder (1999) and Harrell and Martin (2015).

**5. Premaxilla, predental rostrum, foramina size:** large (0); small, pinhole-like (1).

**Exemplars:** FHSM VP-2295 (0), FHSM VP-7262 (1). Some specimens, e.g. FMNH P15144, have both state 0 and state 1.

**Note:** This character is figured in Figure S2. Large foramina on the premaxillary rostrum is purportedly diagnostic for *T. kansasensis* (Everhart, 2005) and was not considered to be ontogenetically variable by Jiménez-Huidobro, Simões, and Caldwell (2016).

**6. Premaxilla, premaxilla-maxilla suture terminus:** anterior to the fourth maxillary tooth (0), at or posterior to the fourth maxillary tooth.

**Exemplars:** FHSM VP-2295 (0), FHSM VP-3 (1).

**Note:** This is a phylogenetic character from Bell (1997; character number 36); state 0 is diagnostic of *T. nepaeolicus* and state 1 is diagnostic of *T. proriger*.

**7. Premaxilla, premaxilla-maxilla suture shape, lateral view:** rectangular (0), u-shaped (1), m-shaped (2).

**Exemplars:** RMM 5610 (0), FHSM VP-3 (1), FMNH P15144 (2).

**Note:** This character is figured in Figure S2. Variation of this character was identified by Jiménez-Huidobro, Simões, and Caldwell (2016) in *T. proriger,* but was considered ontogenetically uninformative.

**Maxilla (2 characters)**

**8. Maxilla, tooth count:** 20-24 (0), 17-19 (1), 15-16 (2), 14 (3), 13 (4), 12 (5).

**Exemplars:** FHSM VP-3 (4), FHSM VP-7262 (5). No specimen in this project exhibits

states 0, 1, 2, or 3.

**Note:** This is a phylogenetic character from Bell (1997, character number 35), and has been used to diagnose *T, proriger*, *T. kansasensis*, and *T. nepaeolicus* (Everhart, 2005; Jiménez-Huidobro & Caldwell, 2019).

**9. Maxilla, tooth row, length between first and sixth maxillary teeth to TSL:** less than 25% (0), greater than or equal to 25% (1).

**Exemplars:** FHSM VP-3 (0), KUVP 66129 (1).

**Note:** Length between the teeth was measured starting at the anterior edge of the base of the first tooth and ending at the posterior edge of the base of the sixth tooth. Total skull length was measured dorsally from the tip of the premaxillary rostrum to the posterior edge of the occipital condyle.

**Quadrate (18 characters)**

**10. Quadrate, quadrate height:** less than 50 mm (0), 50 – 99 mm (1), 100 – 149 mm (2), 150 – 199 mm (3), greater than or equal to 200 mm (4).

**Exemplars:** FHSM VP-14845 (0), FMNH UR902 (1), KUVP 1033 (2), FHSM VP-3 (3), KUVP 5033 (4).

**Note:** Quadrate height was measured as the distance between the bottommost point of the mandibular condyle to the topmost point of the suprastapedial process.

**11. Quadrate, suprastapedial process length, lateral view:** short, ends above the midpoint of the bone (0); intermediate, ends at about mid-height of the bone (1); long, ends distinctly beyond mid-height of the bone (2).

**Exemplars:** FHSM VP-9350 (0), FMNH P 15144 (1), FHSM VP-78 (2).

**Note:** This character is figured in Figure S2. It is a phylogenetic character from Bell (1997; character number 44); state 1 diagnoses *T. proriger* and *T. kansasensis/ nepaeolicus* (Jiménez-Huidobro & Caldwell, 2019).

**12. Quadrate, suprastapedial process thickness, lateral view:** slender (0), robust (1).

**Exemplars:** RMM 5610 (0), FHSM VP-3 (1).

**Note:** This character is figured in Figure S2. It was identified as ontogenetically variable in *T. proriger* and *T. kansasensis/nepaeolicus* by Jiménez-Huidobro, Simões, and Caldwell (2016).

**13. Quadrate, suprastapedial process medial curvature, posterior view:** distinct medial curvature of the suprastapedial process present (0), absent (1).

**Exemplars:** FHSM VP-14845 (0), FHSM VP-3 (1).

**Note:** This character is figured in Figure S2. It is a phylogenetic character from Bell (1997; character number 45). State 1 purportedly diagnoses *T. kansasensis* (Everhart, 2005).

**14. Quadrate, suprastapedial process constriction, view unclear:** present (0), absent (1).

**Exemplars:** unclear.

**Note:** This is a phylogenetic character from Bell (1997; character number 45), and state 1 purportedly diagnoses *T. kansasensis*. This character was not coded in this project for any specimen because the author could not identify either state with certainty.

**15. Quadrate, infrastapedial process development:** absent (0), present (1).

**Exemplars:** FHSM VP-78 (0), FMNH P15144 (1).

**Note:** This character is figured in Figure S2. It is a phylogenetic character from Bell (1997; character number 49). *T. kansasensis/nepaeolicus* is purportedly diagnosed by either state 0 or state 1, and *T. proriger* is diagnosed by state 1 (Jiménez-Huidobro & Caldwell, 2019).

**16. Quadrate, infrastapedial process shape, lateral view:** broadly pointed (0); expanded, rounded, semicircular (1).

**Exemplars:** FMNH UR902 (0), FMNH P15144 (1).

**Note:** This character is figured in Figure S2. Variation in this character was identified by Stewart and Mallon (2018) in *T. proriger* but was considered ontogenetically uninformative.

**17. Quadrate, quadrate height to TSL:** less than 13% (0), greater than or equal to 13% (1).

**Exemplars:** FHSM VP-78 (0), FHSM VP-2295 (1).

**Note:** Quadrate height was measured as the distance between the bottommost point of the mandibular condyle to the topmost point of the suprastapedial process.

**18. Quadrate, tympanic ala thickness:** thin (0), thick (1).

**Exemplars:** AMNH FARB 4909 (0), FMNH UR902 (1).

**Note:** This is a phylogenetic character from Bell (1997; character number 51). State 0 purportedly diagnoses *T. proriger,* and state 1 diagnoses *T. kansasensis/nepaeolicus* (Everhart, 2005; Jiménez-Huidobro & Caldwell, 2019).

**19. Quadrate, tympanic ala concavity:** deep (0), shallow (1).

**Exemplars:** AMNH FARB 4909 (0), FMNH UR902 (1).

**Note:** This is a phylogenetic character from Bell (1997; character number 52). State 1 is purportedly diagnostic of *T. kansasensis* (Everhart, 2005).

**20. Quadrate, tympanic ala, anteroventral corner, lateral view:** absent (0), present (1).

**Exemplars:** All specimens in this project exhibit state 0.

**Note:** This character is figured in Figure S2. It is a phylogenetic character from Bell (1997; character number 54); state 1 was identified by Street and Caldwell (2017) as diagnostic for *Mosasaurus hoffmannii*. It was not previously considered to be ontogenetically variable.

**21. Quadrate, tympanic ala lateral rim distinction:** absent or weak (0), distinct ridge present (1).

**Exemplars:** FHSM VP-78 (0), AMNH FARB 1555 (1).

**Note:** This character was identified in *Tethysaurus nopcsai* by Houssaye and Bardet (2013).

**22. Quadrate, tympanic ala lateral crest end position:** unclear.

**Exemplars:** unclear.

**Note:** This character–specifically, a “lateral crest of tympanic ala” that “ends posteriorly near mandibular condyle”–is phylogenetic and was considered diagnostic for *T. nepaeolicus* by Jiménez-Huidobro and Caldwell (2019). This character was not coded in this project for any specimen because the author could not identify it or its states with certainty.

**23. Quadrate, medial ridge, ventral diversion, medial view:** single thin ridge (0); thin ridge that diverges ventrally (1).

**Exemplars:** unclear.

**Note:** This is a phylogenetic character from Bell (1997; character number 60). *T. kansasensisis* is described as possessing state 1, but it is not diagnosed by it (Everhart, 2005). This character was not coded in this project for any specimen because the author could not identify either of its states with certainty.

**24. Quadrate, stapedial pit distinction, medial view:** absent (0); distinct ridge present, edge of pit well-defined (1).

**Exemplars:** FHSM VP-14848 (0), AMNH FARB 1555 (1).

**Note:** This character was identified in *Tethysaurus nopcsai* by Houssaye and Bardet (2013).

**25. Quadrate, mandibular condyle ossification, ventral view:** mandibular condyle spongy (0), mandibular condyle completely ossified (1).

**Exemplars:** FHSM VP-14848 (0), FMNH P15144 (1).

**Note:** This character was identified in *Tethysaurus nopcsai* by Houssaye and Bardet (2013).

**26. Quadrate, mandibular condyle, anterodorsal deflection, lateral view:** distinct anterodorsal deflection of mandibular condyle present (0); absent, mandibular condyle rounded (1).

**Exemplars:** RMM 5610 (0), AMNH FARB 4909 (1).

**Note:** This character is figured in Figure S2. It was identified by the author and is similar to a phylogenetic character from Bell (1997; character number 63), but the ontogenetic character states are backwards (i.e., according to Bell [1997], state 0 is absent and state 1 is present).

**27. Quadrate, mandibular condyle width, view unclear:** narrow (0), broad (1).

**Exemplars:** unclear.

**Note:** This is a phylogenetic character from Jiménez-Huidobro and Caldwell (2019), where state 1 is diagnostic of *T. nepaeolicus.* It is potentially the same as character number 62 from Bell (1997), in which case the ontogenetic character states are backwards (i.e., according to Bell [1997], state 0 is broad and state 1 is narrow). This character was not coded in this project for any specimen because the author could not identify either of its states with certainty.

**Frontal (5 characters)**

**28. Frontal, shape, dorsal view:** narrow triangle (0), broad diamond/kite (1).

**Exemplars:** KUVP 28705 (0), KUVP 1032 (1).

**Note:** This character was identified by Harrell and Martin (2015) in *Mosasaurus hoffmannii*.

**29. Frontal, frontal-parietal suture complexity, dorsal view:** mostly straight, smooth (0); convoluted (1).

**Exemplars:** All specimens in this project exhibit state 0.

**Note:** This character was identified by Harrell and Martin (2015) in *Mosasaurus hoffmannii*. It does not appear to vary in *T. proriger* or *T. kansasensis/nepaeolicus* to the same extent that it does in *M. hoffmannii*.

**30. Frontal, posterolateral process shape, dorsal view:** slender (0), robust (1).

**Exemplars:** RMM 5610 (0), KUVP 65636 (1).

**Note:** This character is figured in Figure S2. It was identified in *T. proriger* and *T. kansasensis/nepaeolicus* by Jiménez-Huidobro, Simões, and Caldwell (2016); it is also a phylogenetic character from Bell (1997; character number 13).

**31. Frontal, dorsal midline crest:** absent (0), present (1).

**Exemplars:** AMNH FARB 124/134 (0), KUVP 66129 (1).

**Note:** This was identified in *T. proriger* and *T. kansasensis/nepaeolicus* by Jiménez-Huidobro, Simões, and Caldwell (2016); it is also a phylogenetic character from Bell (1997; character number 12). *T. nepaeolicus* is purportedly diagnosed by state 0, and *T. proriger* and *T. kansasensis* are diagnosed by state 1 (Everhart, 2005; Jiménez-Huidobro & Caldwell, 2019).

**32. Frontal, frontal-parietal suture, medial flange size, dorsal view:** small (0), large (1).

**Exemplars:** FHSM VP-2295 (0), AMNH FARB 124/134 (1).

**Note:** This character is figured in Figure S2. It is a phylogenetic character from Bell (1997; character number 20), and *T. kansasensis* is purportedly diagnosed by state 1.

**Prefrontal (2 characters)**

**33. Prefrontal, prefrontal-postorbitofrontal overlap:** ventral overlap present (0), lateral overlap present (1).

**Exemplars:** unclear.

**Note:** This is a phylogenetic character from Bell (1997; character number 30). According to Jiménez-Huidobro and Caldwell (2019), both *T. proriger* and *T. nepaeolicus* are diagnosed by a “prefrontal that overlaps with postorbitofrontal;” however, in mosasaurs, the phylogenetically relevant differences are in overlap position and not overlap presence (Bell, 1997). This character was not coded in this project for any specimen because the author could not identify either of its states with certainty.

**34. Prefrontal, vaulting, anterior view*:*** absent (0), present (1)

**Exemplars:** unclear.

**Note:** This character was identified by Bardet, Suberbiola, and Jalil (2003); state 1 is diagnostic of *Tethysaurus nopcsai*. This character was not coded in this project for any specimen because the author could not identify either of its states with certainty.

**Parietal (5 characters)**

**35. Parietal, posterior pegs over supraoccipital, dorsal view:** absent (0), two bumps on parietal dorsal to supraoccipital present and small (1), bumps large and pointed (2).

**Exemplars:** FMNH P15144 (0), RMM 5610 (1). No specimen in this project exhibits

state 2.

**Note:** This character is figured in Figure S2. It was identified by Bardet, Suberbiola, and Jalil (2003); state 2 is diagnostic of *Tethysaurus nopcsai*.

**36. Parietal, dorsal, medial, and lateral invasion by the frontal, dorsal view:** absent (0), parietal invaded medially and laterally by the frontal (1).

**Exemplars:** unclear.

**Note:** This is a phylogenetic character from Bell (1997; character number 19), and state 1 purportedly diagnoses *T. proriger* (Jiménez-Huidobro & Caldwell, 2019). This character was not coded in this project for any specimen because the author could not identify either of its states with certainty.

**37. Parietal, nuchal fossa, dorsal view:** fossa absent, posterodorsal surface of parietal flat (0); fossa present (1).

**Exemplars:** YPM 3974 (0), FHSM VP-2295 (1).

**Note:** This character is figured in Figure S2. It was identified as ontogenetically variable in *T. proriger* and *T. kansasensis/nepaeolicus* by Jiménez-Huidobro, Simões, and Caldwell (2016).

**38. Parietal, lateral borders, dorsal view:** lateral border convex (0), lateral borders straight (1).

**Exemplars:** FHSM VP-2295 (0), AMNH FARB 124/134 (1).

**Note:** This character is figured in Figure S2. It is a phylogenetic character; state 1 purportedly diagnoses *T. nepaeolicus* (Jiménez-Huidobro & Caldwell, 2019).

**39. Parietal, parietal foramen position:** far from frontal-parietal suture (0), close to frontal-parietal suture (1), bordering frontal-parietal suture or invading frontal (2).

**Exemplars:** CMN 8162 (1), FHSM VP-15632 (2). No specimen in this project exhibits

state 0.

**Note:** This is a phylogenetic character from Bell (1997; character number 24), and state 2 purportedly diagnoses *T. kansasensis* (Everhart, 2005).

**Jugal (3 characters)**

**40. Jugal, posteroventral process, lateral view:** absent (0), present (1).

**Exemplars:** CMN 8162 (0), FMNH P15144 (1).

**Note:** This character is figured in Figure S2. It is a phylogenetic character from Bell (1997; character number 40). Jiménez-Huidobro, Simões, and Caldwell, 2016 noted that the position of the process is variable; in the juvenile RMM 5610 and some adults (FHSM VP-3) the process is at the bottom of the vertical ramus, whereas in other adults (FMNH P15144, FFHM 1997-10) the process is at the intersection of the vertical and horizontal rami.

**41. Jugal, posteroventral angle, lateral view:** obtuse (0), nearly 90 degrees (1).

**Exemplars:** RMM 5610 (0), FHSM VP-7262 (1).

**Note:** This is a phylogenetic character from Bell (1997; character number 39), but its coding for ontogeny is binary (i.e., according to Bell [1997], state 0 is very obtuse or curvilinear, state 1 is slightly obtuse, and state 2 is nearly 90 degrees). State 1 is purportedly diagnostic for *T. kansasensis* (Everhart, 2005).

**42. Jugal, ascending ramus, thickness, lateral view:** slender, narrow (0); robust, expanded dorsally (1).

**Exemplars:** RMM 5610 (0), FHSM VP-7262 (1).

**Note:** This character was identified by Bardet, Suberbiola, and Jalil (2003) in *Tethysaurus nopcsai*.

**Pterygoid (3 characters)**

**43. Pterygoid, tooth count:** more than 14 (0), less than or equal to 14 (1).

**Exemplars:** AMNH FARB 4909 (1). No specimen in this project exhibits state 0.

**Note:** Although this character is included in diagnoses of mosasaurs and varies across taxa, it has apparently not been included in phylogenetic analyses of mosasaur relationships (i.e., its states were not described by Bell [1997] or Jiménez-Huidobro and Caldwell [2019]). The states were chosen based on the basal position of the russelosaurine *Tethysaurus nopcsai*, which has “15 to 19 pterygoid teeth” (Bardet, Suberbiola, & Jalil, 2003) relative to *Tylosaurus*, which generally has less than 15 pterygoid teeth (Jiménez-Huidobro & Caldwell, 2019). The potential for this character to be ontogenetically variable or sexually dimorphic in mosasaurs, as it is in some extant lizards (Skawiński, Borczyk, & Turniak, 2017), has not been previously tested.

**44. Pterygoid, ectopterygoid process, contact with maxilla:** present (0), absent (1).

**Exemplars:** unclear.

**Note:** This is a phylogenetic character from Bell (1997; character number 41), and state 1 diagnoses *T. kansasensis* and *T. nepaeolicus* (Everhart, 2005; Jiménez-Huidobro & Caldwell, 2019). This character was not coded in this project for any specimen because the author could not identify either of its states with certainty.

**45. Pterygoid, ectopterygoid process, shape, dorsal and ventral views:** slender (0), wide and flat (1).

**Exemplars:** AMNH FARB 4909 (0), FHSM VP-3 (1).

**Note:** This character is figured in Figure S2. It was identified by Houssaye and Bardet (2013) in *Tethysaurus nopcsai*.

**Basioccipital (3 characters)**

**46. Basioccipital, shape, posterior view:** circular to semicircular (0), reniform (1).

**Exemplars:** unclear.

**Note:** This character was identified by Houssaye and Bardet (2013) in *Tethysaurus nopcsai*. This character was not coded in this project for any specimen because the author could not identify either of its states consistently.

**47. Basioccipital, occipital condyle, ossification, posterior view:** occipital condyle spongy (0), occipital condyle completely ossified (1).

**Exemplars:** AMNH FARB 1585 (0), FHSM VP-15632 (1).

**Note:** This character was identified by Houssaye and Bardet (2013) in *Tethysaurus nopcsai*.

**48. Basioccipital, medullar floor foramina:** unclear.

**Exemplars:** unclear.

**Note:** This character identified by Bardet, Suberbiola, and Jalil (2003); *Tethysaurus nopcsai* is diagnosed by a “medullar floor of the basioccipital pierced by three foramina.” This character was not coded in this project for any specimen because the author could not identify it or its states with certainty.

**Dentary (6 characters)**

**49. Dentary, dentary depth, lateral view:** slender, more than six times longer than tall (0); deep, less than or equal to six times longer than tall (1).

**Exemplars:** KUVP 66129 (0), FHSM VP-3 (1).

**Note:** This character was identified by Stewart and Mallon (2018), but the authors did not specify exact values for either state. In this project, the exact ratio of dentary length to dentary height was calculated. Dentary length was measured along the ventral edge of the bone, and dentary height was measured as the tallest dorsoventral distance on the posterior end of the bone. When the exact dentary length and/or height was not provided and could not be estimated using scale bars, the ratio was estimated by placing calipers to a photograph of the specimen and measuring the relative length and height.

**50. Dentary, predental process, dorsal ridge, lateral view:** absent (0); present (1).

**Exemplars:** AMNH FARB 4909 (0), FMNH P15144 (1).

**Note:** This character is figured in Figure S2. It was identified by the author.

**51. Dentary, tooth row, length between first and sixth dentary teeth to TSL:** greater than 23% (0), less than or equal to 23% (1).

**Exemplars:** FHSM VP-78 (0), FHSM VP-2295 (1).

**Note:** Length between the teeth was measured starting at the anterior edge of the base of the first tooth and ending at the posterior edge of the base of the sixth tooth. Total skull length was measured rostrocaudally from the tip of the premaxillary rostrum to the posterior edge of the occipital condyle.

**52. Dentary, tooth row, length between first and sixth dentary teeth to dentary length:** greater than 35% (0), less than or equal to 35% (1).

**Exemplars:** KUVP 1032 (0), FHSM VP-3 (1).

**Note:** Length between the teeth was measured starting at the anterior edge of the base of the first tooth and ending at the posterior edge of the base of the sixth tooth. Dentary length was measured along the ventral edge of the bone.

**53. Dentary, dentary length to lower jaw length:** greater than 60% (0), between 60% and 56% (1), less than or equal to 55% (2).

**Exemplars:** FHSM VP-2295 (0), FHSM VP-2495 (1).

**Note:** Dentary length was measured along the ventral edge of the bone, and lower jaw length was measured laterally from the anterior end of the dentary to the posterior edge of the articular.

**54. Dentary, tooth count:** 20-24 (0), 17-19 (1), 15-16 (2), 14 (3), 13 (4), 12 (5), < 12 (6)

**Exemplars:** FHSM VP-15632 (2), AMNH FARB 124/134 (3), CMN 8162 (4). No specimens in this project exhibit states 0, 1, 5, or 6.

**Note:** This is a phylogenetic character from Bell (1997; character number 68) and is included in the diagnoses of both species in this project (Jiménez-Huidobro & Caldwell, 2019). Variation in this character has been noted in *T. kansasensis/nepaeolicus*, but not *T. proriger*.

**Splenial (1 character)**

**55. Splenial, dorsomedial process notch:** notch absent (0), present (1).

**Exemplars:** unclear.

**Note:** This character was identified by Bardet, Suberbiola, and Jalil (2003), and state 1 diagnoses *Tethysaurus nopcsai*. This character was not coded for many specimens in this project because splenials are often either missing, not described or figured in the literature, or fragmentary.

**Surangular (1 character)**

**56. Surangular, medial exposure ventral to coronoid:** absent (0), present (1).

**Exemplars:** unclear.

**Note:** This character was identified by Bardet, Suberbiola, and Jalil (2003), and state 1 diagnoses *Tethysaurus nopcsai*. This character was not coded in this project for any specimen because the author could not identify it or its states with certainty.

**Coronoid (2 characters)**

**57. Coronoid, anterolateral flange, notch:** notch absent (0), notch shallow, broad (1), notch acute, deeply c-shaped (2).

**Exemplars:** AMNH FARB 1565 (0), FHSM VP-2209 (1). No specimen in this project exhibits state 2.

**Note:** This character is figured in Figure S2. It was identified by Harrell and Martin (2015) in *Mosasaurus hoffmannii*.

**58. Coronoid, posteroventral process:** absent (0), present as bump (1), fan-like (2).

**Exemplars:** CMN 51258-51263 (0), RMM 5610 (1), FMNH P15144 (2).

**Note:** This character is figured in Figure S2. It was identified by Konishi, Jiménez-Huidobro, and Caldwell (2018) in *T. proriger*.

**Marginal Dentition (1 character)**

**59. Teeth, fluting:** absent (0), present (1).

**Exemplars:** unclear.

**Note:** This is a phylogenetic character from Bell (1997; character number 86), and state 9 diagnoses *T. proriger*. This character was not coded in this project for any specimen because the author could not identify it or its states with certainty.

**References**

Bardet N, Suberbiola XP, Jalil NE. 2003. A new mosasauroid (Squamata) from the Late

Cretaceous (Turonian) of Morocco. Comptes Rendus Palevol 2**:**607-616.

Bell GL. 1997. A phylogenetic revision of North American and Adriatic Mosasauroidea; pp.

293-332 in J. M. Callaway and E. Nicholls (eds.), Ancient Marine Reptiles. Academic

Press, Cambridge, Massachusetts.

Everhart MJ. 2005. *Tylosaurus* *kansasensis*, a new species of tylosaurine (Squamata,

Mosasauridae) from the Niobrara Chalk of western Kansas, USA. Netherlands Journal of

Geosciences 84**:**231-240.

Harrell TL, Martin JE. 2015. A mosasaur from the Maastrichtian Fox Hills Formation of the

northern Western Interior Seaway of the United States and the synonymy of *Mosasaurus*

*maximus* with *Mosasaurus hoffmanni* (Reptilia**:** Mosasauridae). Netherlands Journal of

Geosciences 94**:**23-37.

Houssaye A, Bardet N. 2013. A baby mosasauroid (Reptilia, Squamata) from the Turonian of

Morocco–*Tethysaurus* ‘junior’ discovered? Cretaceous Research 46**:**208-215.

Jiménez-Huidobro P, Simões TR, Caldwell MW. 2016. Re-characterization of *Tylosaurus*

*nepaeolicus* (Cope, 1874) and *Tylosaurus kansasensis* Everhart, 2005**:** ontogeny or

sympatry? Cretaceous Research 65**:**68-81.

Jiménez-Huidobro P, Caldwell MW. 2019. A new hypothesis of the phylogenetic relationships

of the Tylosaurinae (Squamata**:** Mosasauroidea). Frontiers in Earth Science 7**:**47.

Konishi T, Jiménez-Huidobro P, Caldwell MW. 2018. The smallest-known neonate individual of

*Tylosaurus* (Mosasauridae, Tylosaurinae) sheds new light on the Tylosaurine rostrum and

heterochrony. Journal of Vertebrate Paleontology 1-11.

Mulder EW. 1999. Transatlantic latest Cretaceous mosasaurs (Reptilia, Lacertilia) from the

Maastrichtian type area and New Jersey. Geologie en Mijnbouw 78**:**281-300.

Skawiński T, Borczyk B, Turniak E. 2017. Variability of pterygoid teeth in three species of

*Podarcis* lizards and the utility of palatal dentition in lizard systematics. Belgian Journal

of Zoology 147(2).

Stewart RF, Mallon J. 2018. Allometric growth in the skull of *Tylosaurus proriger* (Squamata**:**

Mosasauridae) and its taxonomic implications. Vertebrate Anatomy Morphology

Paleontology 6**:**75.

Street HP, Caldwell MW. 2017. Rediagnosis and redescription of *Mosasaurus hoffmannii*

(Squamata**:** Mosasauridae) and an assessment of species assigned to the genus

*Mosasaurus*. Geological Magazine 154**:**521-557.
