# Supplementary material for: Craniofacial ontogeny in Tylosaurinae
Source: PeerJ. 2020 Oct 20;8:e10145. doi: 10.7717/peerj.10145 (PMC7583613; doi:10.7717/peerj.10145)

Test A: Strict consensus ontogram of *Tylosaurus proriger* including only specimens that were studied first-hand.

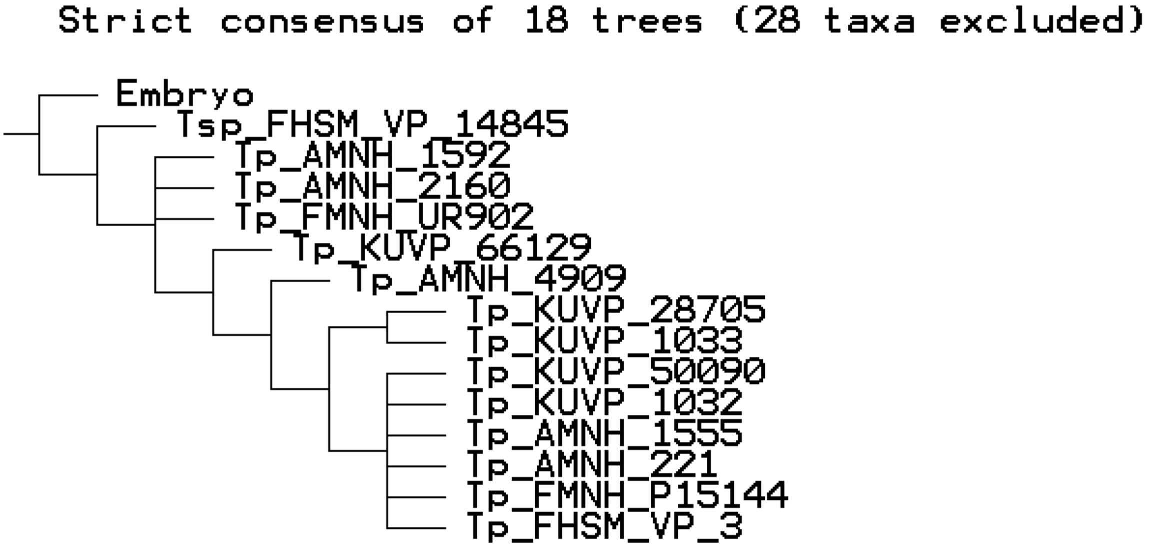

Test B: Ontogram (only one tree recovered) of *Tylosaurus kansasensis/nepaeolicus* including only specimens that were studied first-hand.

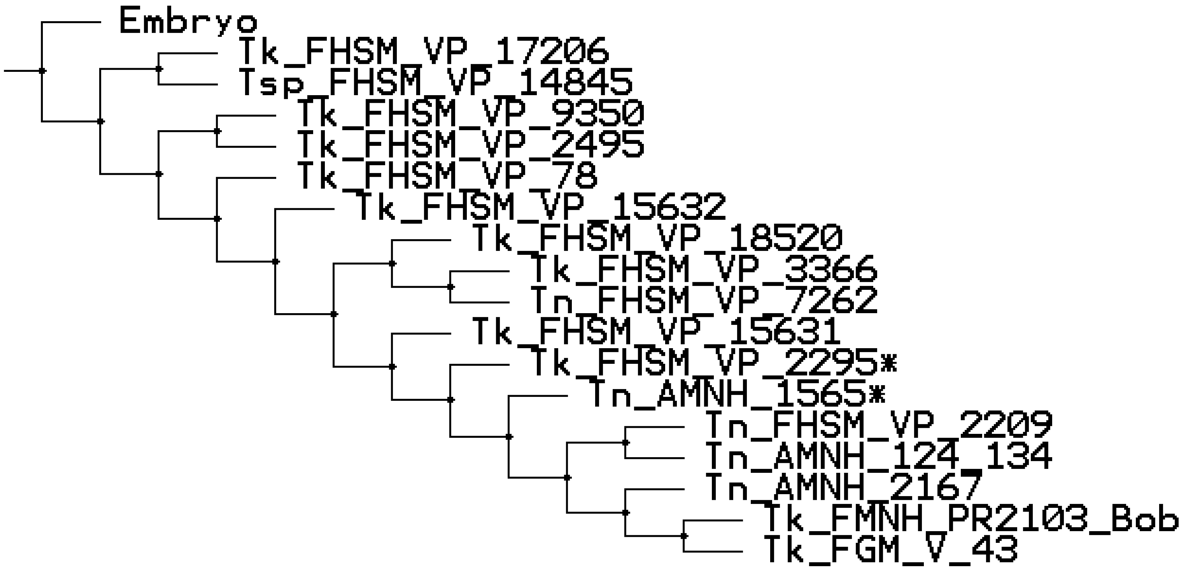

Test C: Strict (top) and 50% majority rule (bottom) ontograms of all three taxa including only specimens that were studied first-hand.

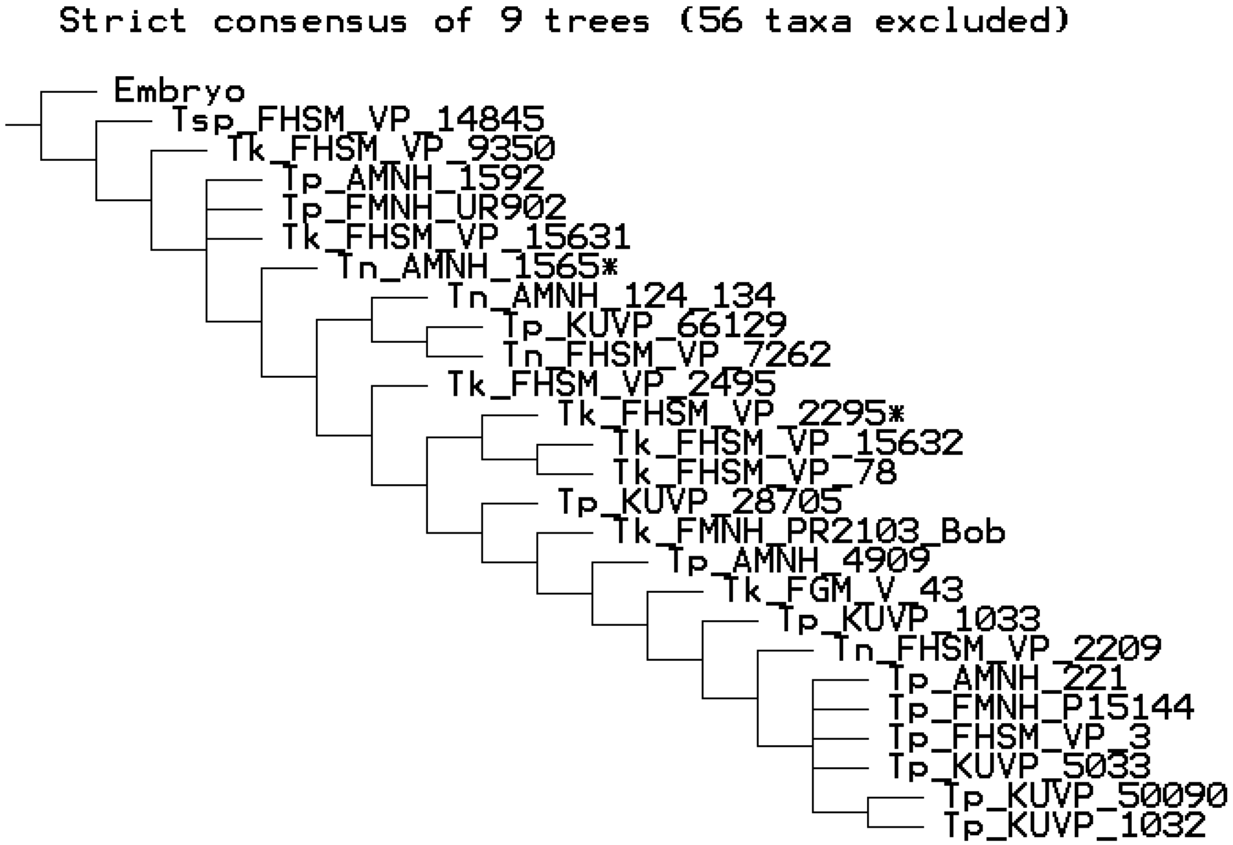

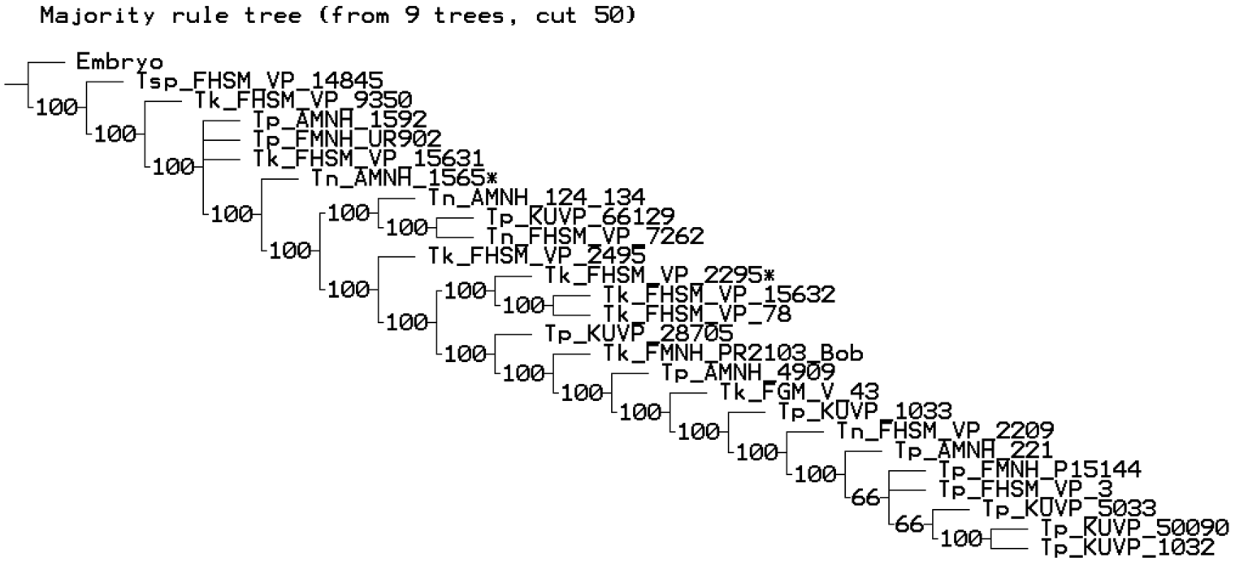

Test D: Strict (top) and 50% majority rule (bottom) ontograms of *Tylosaurus proriger* with size characters (TSL and QH) excluded.

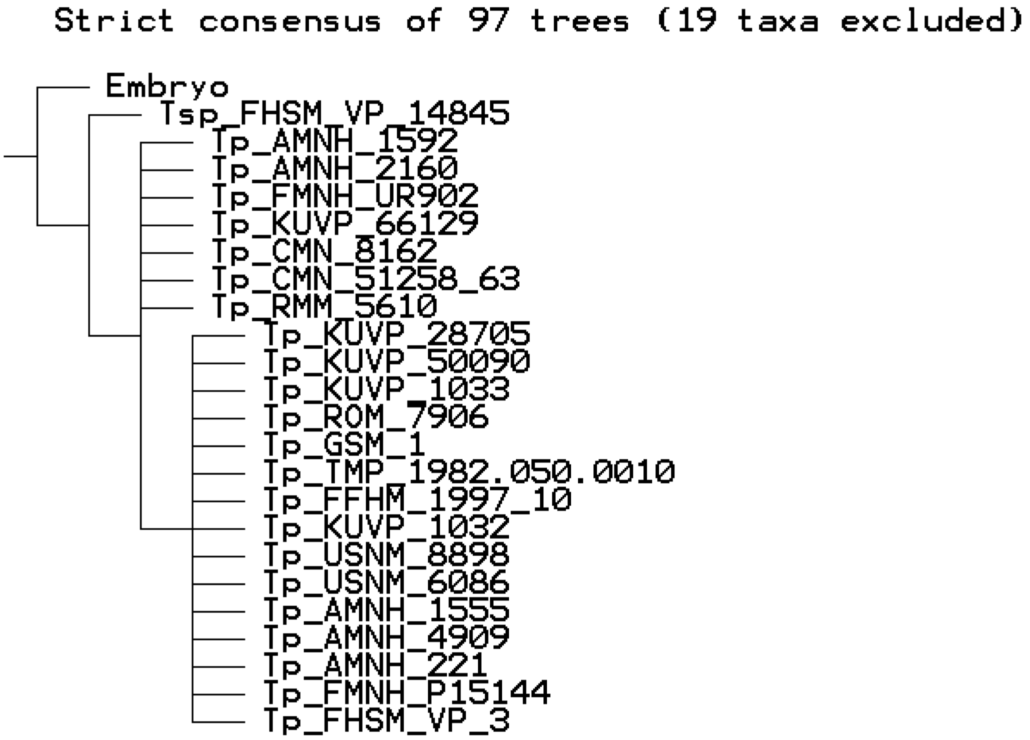

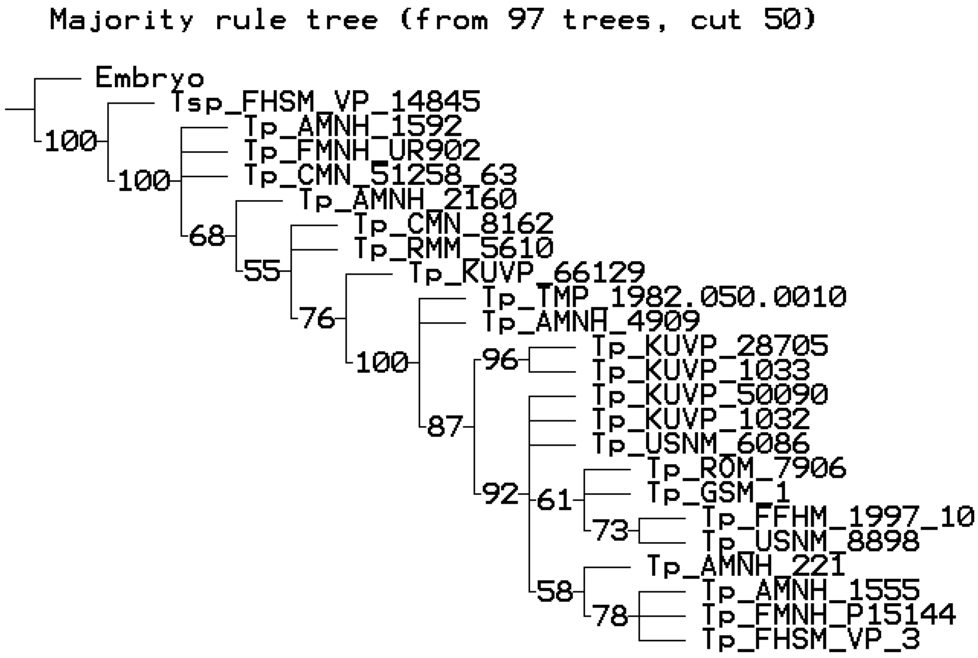

Test E: Strict (top) and 50% majority rule (bottom) ontograms of *Tylosaurus proriger* with size characters (TSL and QH) excluded, including the artificial adult. The artificial adult is recovered as sister to FHSM VP-3 just as it is in the analysis including the size characters.

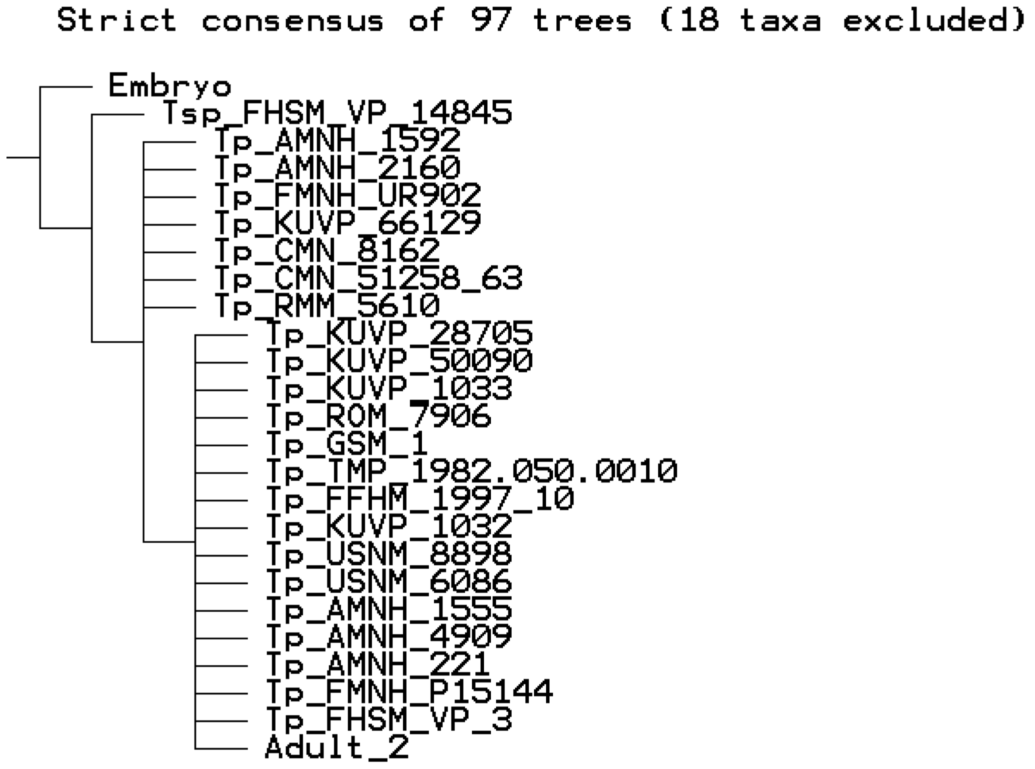

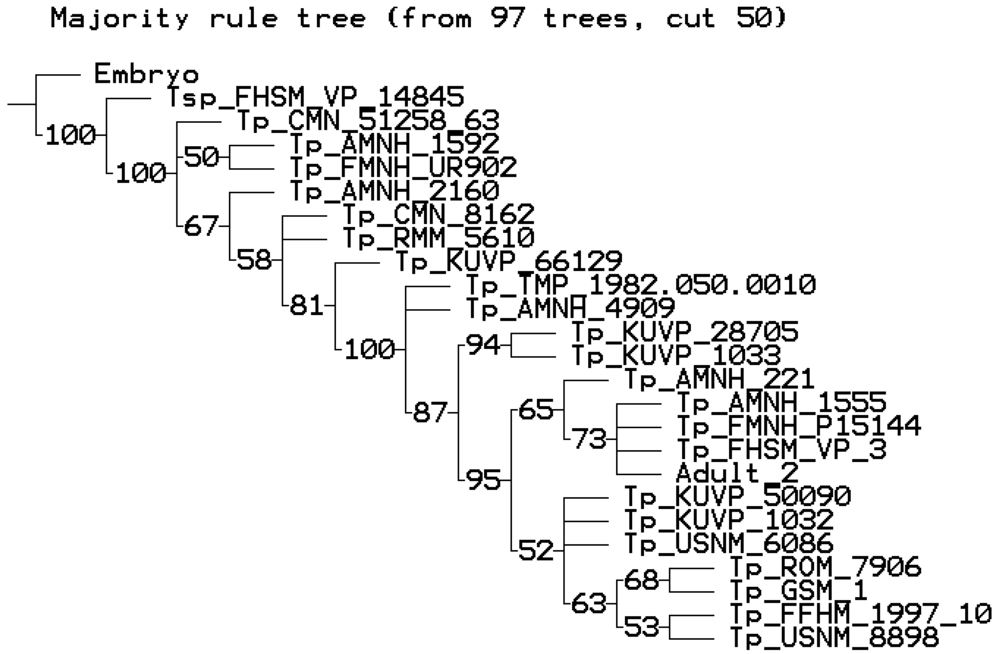

Test F: Strict (top) and 50% majority rule (bottom) ontograms of *Tylosaurus kansasensis/nepaeolicus* with size characters (TSL and QH) excluded.

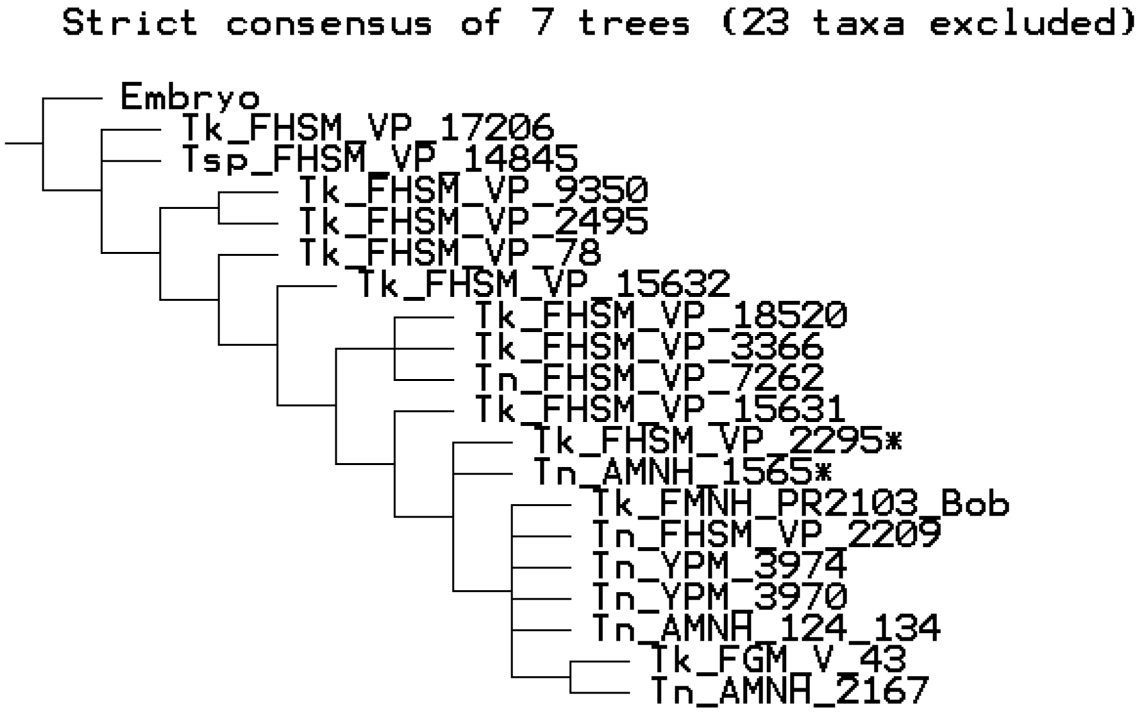

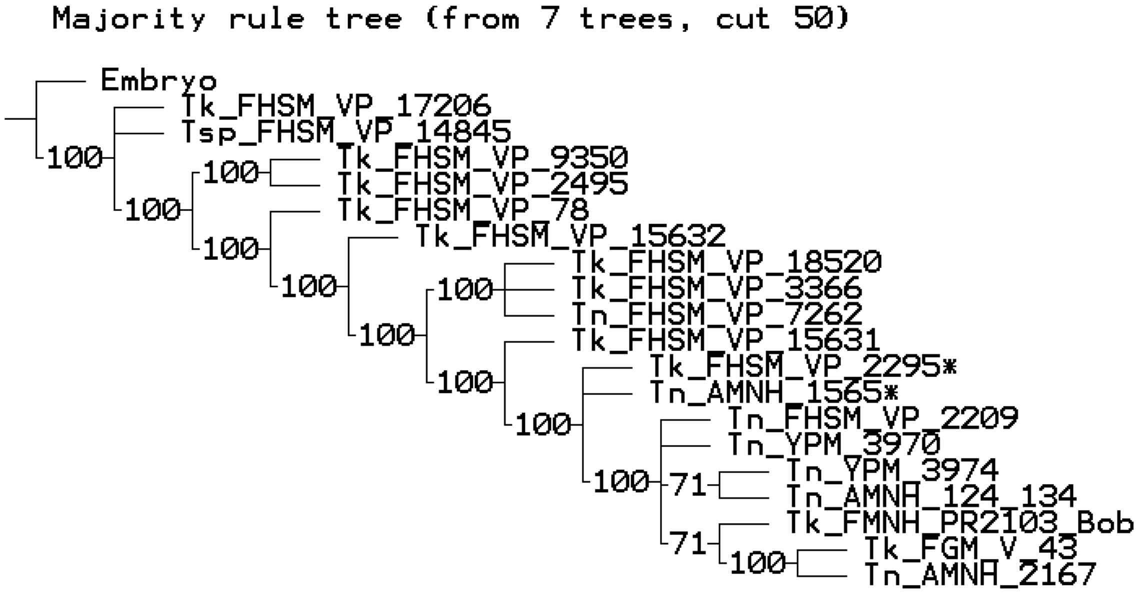

Test G: Strict (top) and 50% majority rule (bottom) ontograms of *Tylosaurus kansasensis/nepaeolicus* with size characters (TSL and QH) excluded, including the artificial adult. The artificial adult is recovered as sister to FHSM VP-2209 and YPM 3970 just as it is in the analysis including the size characters.

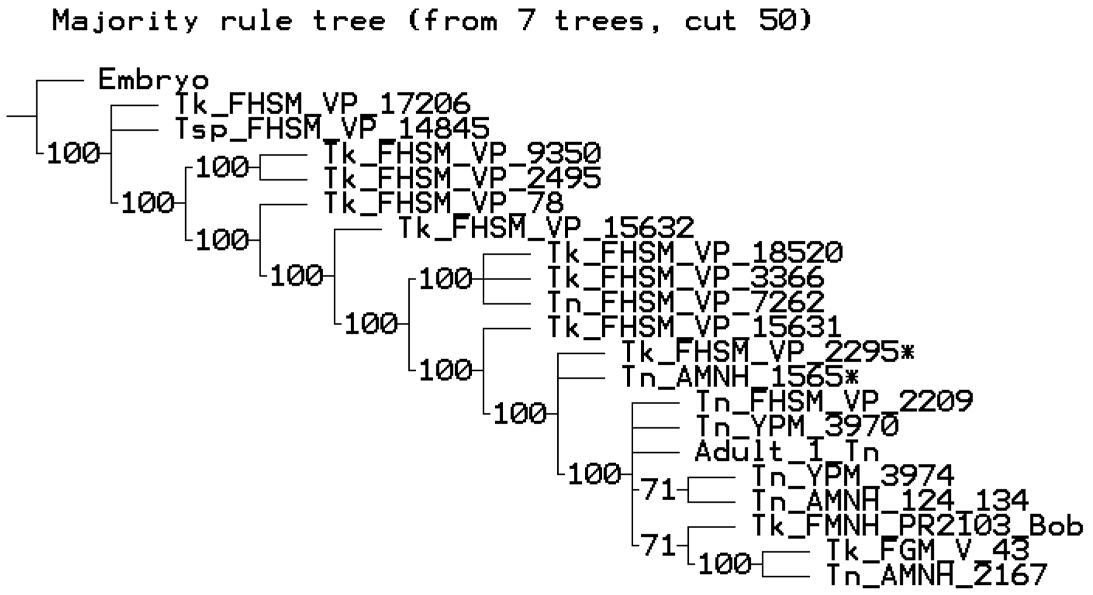

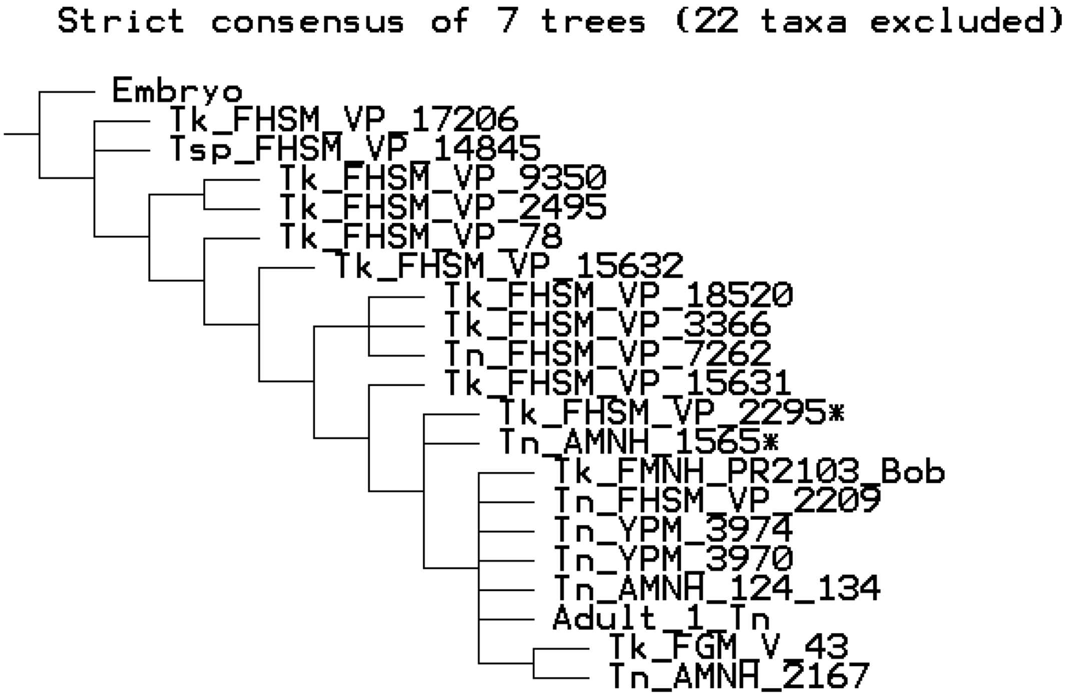

Test H: Strict (top) and 50% majority rule (bottom) ontograms of all three taxa with size characters (TSL and QH) excluded.

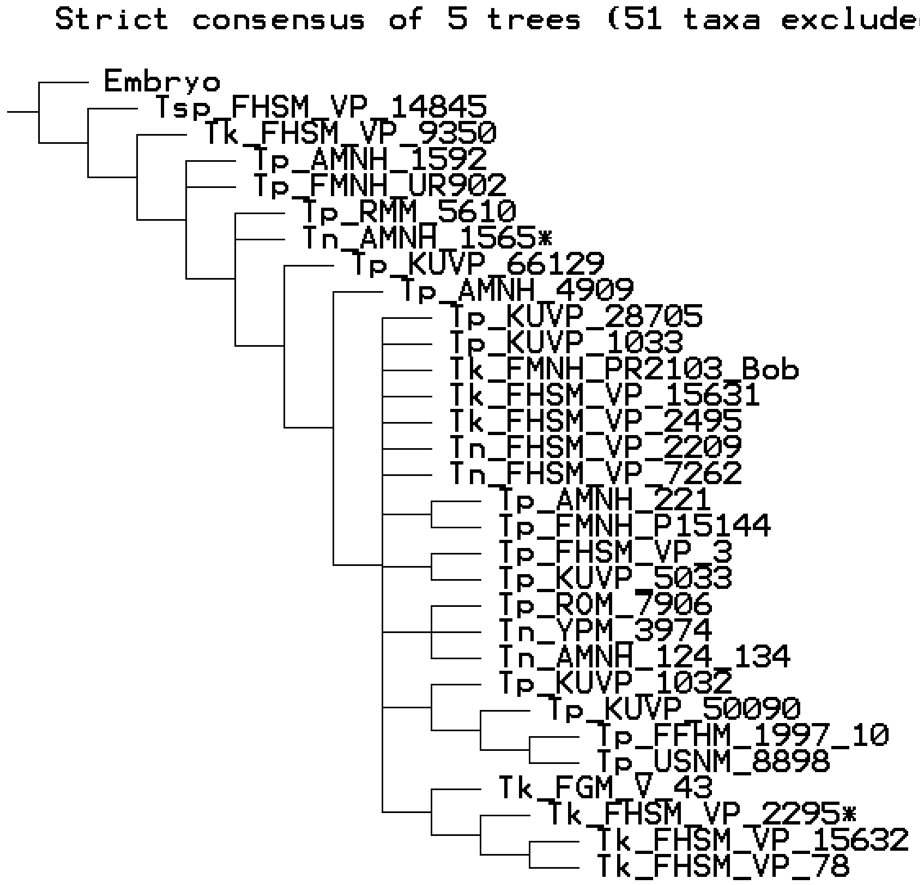

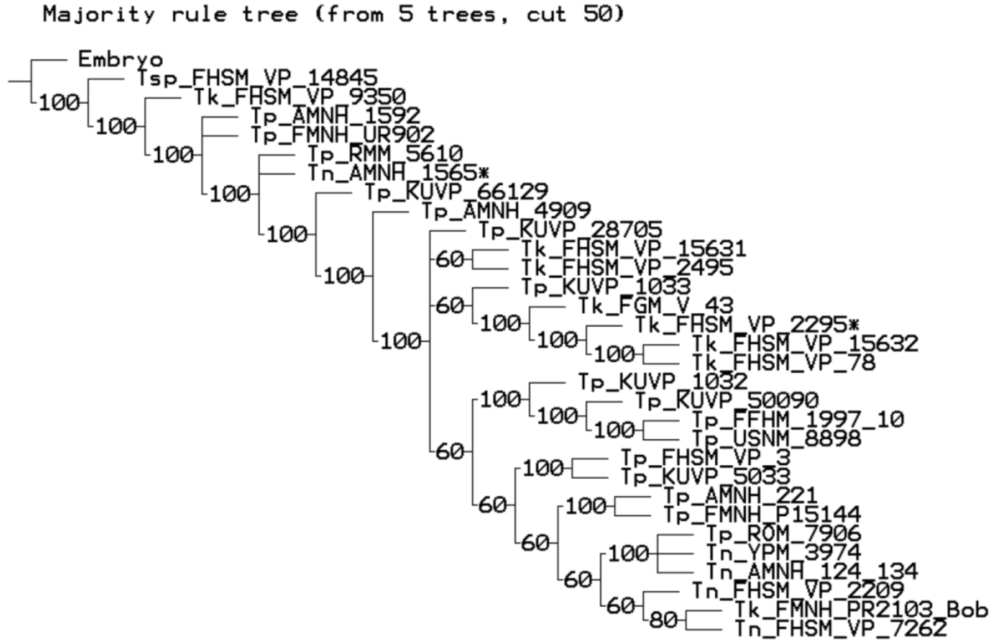

Test I: Strict (top) and 50% majority rule (bottom) ontograms of all three taxa with size characters (TSL and QH) excluded, including the artificial adult. The artificial adult is recovered as sister to the group of relatively mature *T. proriger* (specifically, KUVP 5033 and FHSM VP-3), just as it is in the analysis including the size characters.

Strict consensus of 5 trees (50 taxa excluded)

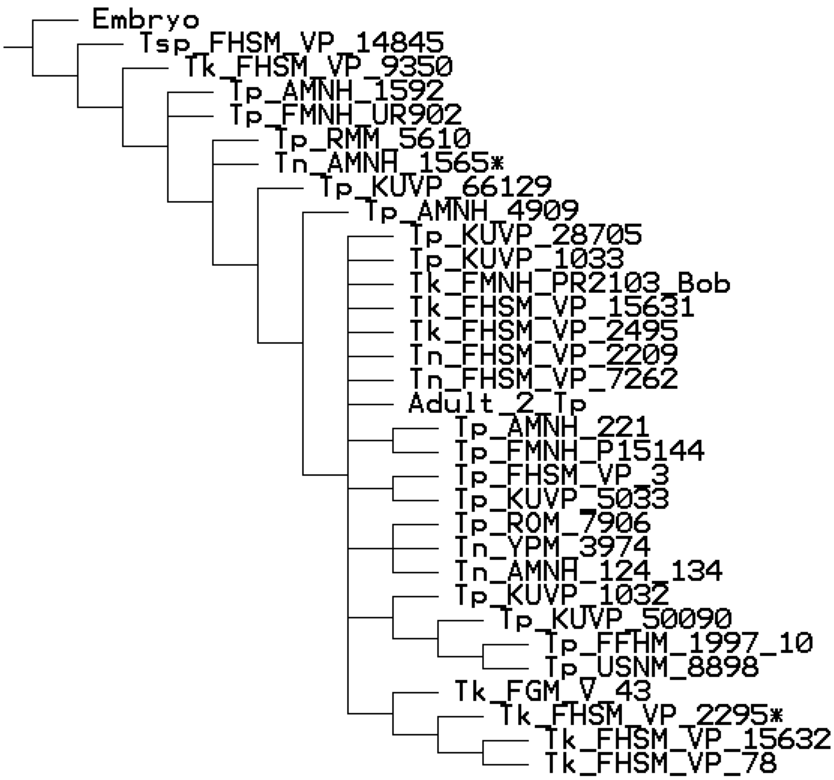

Majority rule tree (from 5 trees, cut 50)

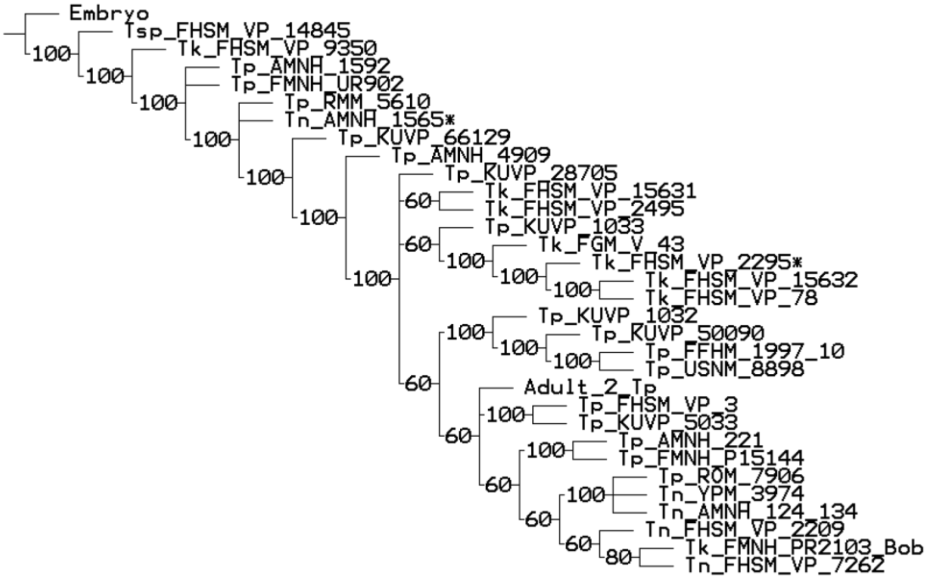

Test J: Strict (top) and 50% majority rule (bottom) ontograms of *Tylosaurus proriger* after TSL states were changed to equal (400 mm) bins. For this analysis, TSL was coded as follows: < 400 mm (0), 400–799 mm (1), 800–1199 mm (2), 1200–1599 mm (3), ≥ 1600 mm (4). Notes: in the original analysis, TSL was coded as follows: < 400 mm (0), 400–799 mm (1), 800–999 mm (2), 1000–1399 mm (3), ≥ 1400 mm (4); the ontogram of *T. kansasensis/nepaeolicus* was not affected by this change, since the largest TSL in that dataset is 890 mm.

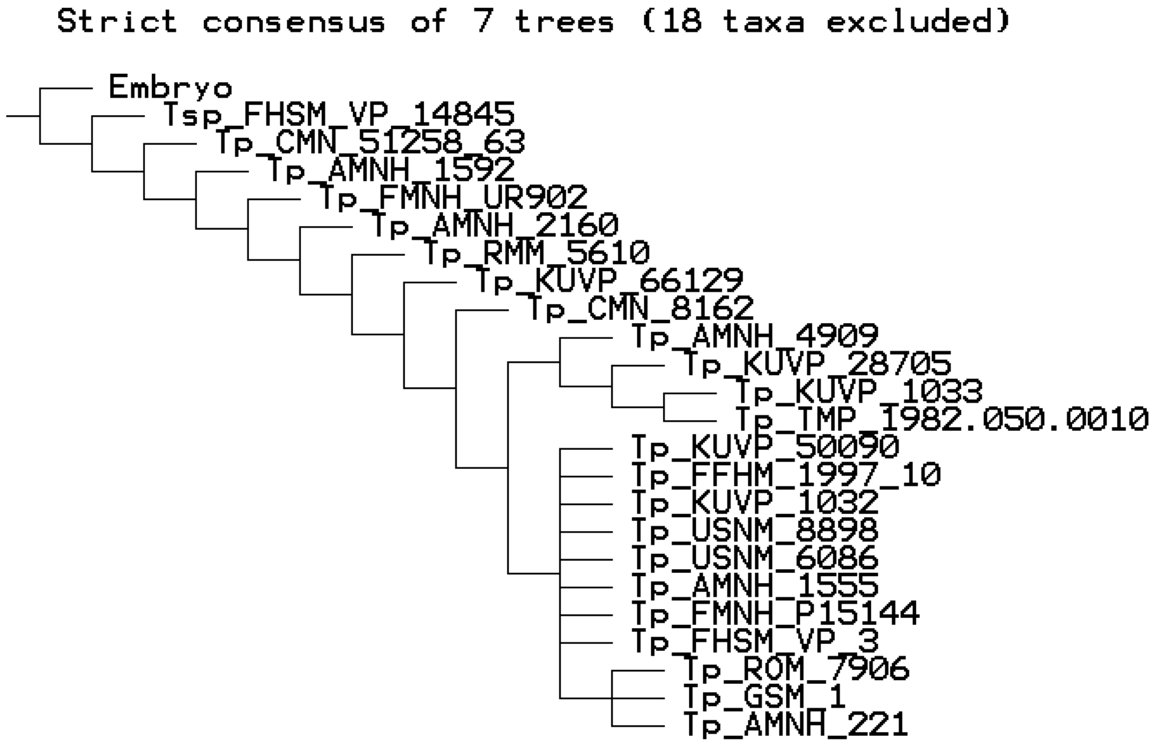

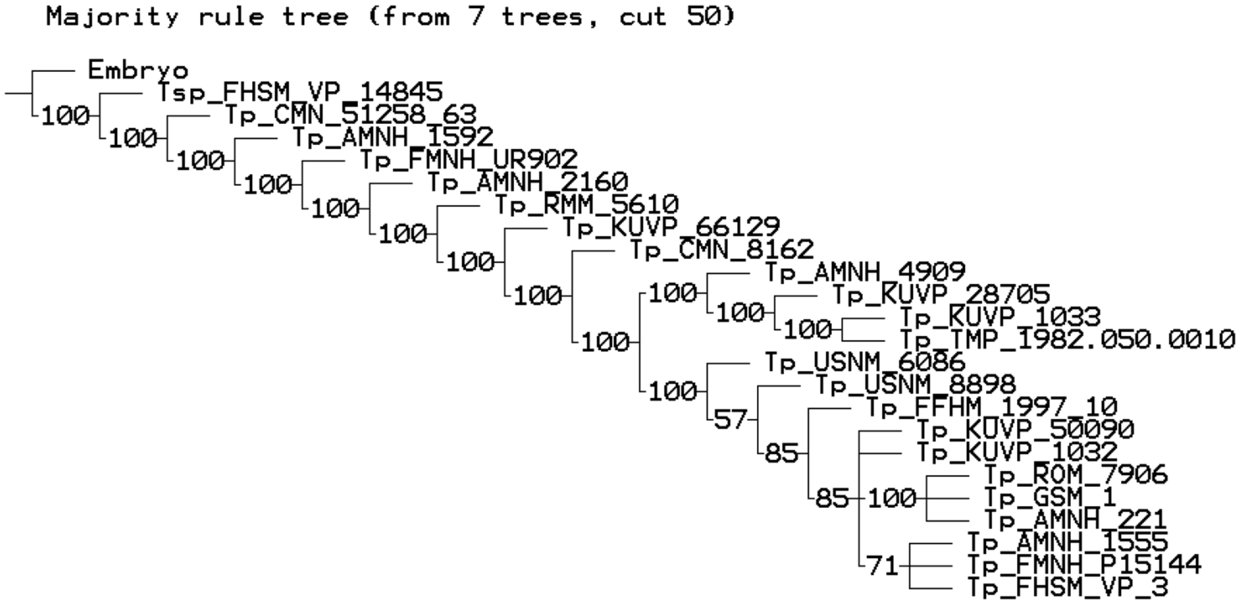

Test K: Strict (top) and 50% majority rule (bottom) ontograms of all three taxa after TSL states were changed to equal (400 mm) bins. For this analysis, TSL was coded as follows: < 400 mm (0), 400–799 mm (1), 800–1199 mm (2), 1200–1599 mm (3), ≥ 1600 mm (4). Note: in the original analysis, TSL was coded as follows: < 400 mm (0), 400–799 mm (1), 800–999 mm (2), 1000–1399 mm (3), ≥ 1400 mm (4).

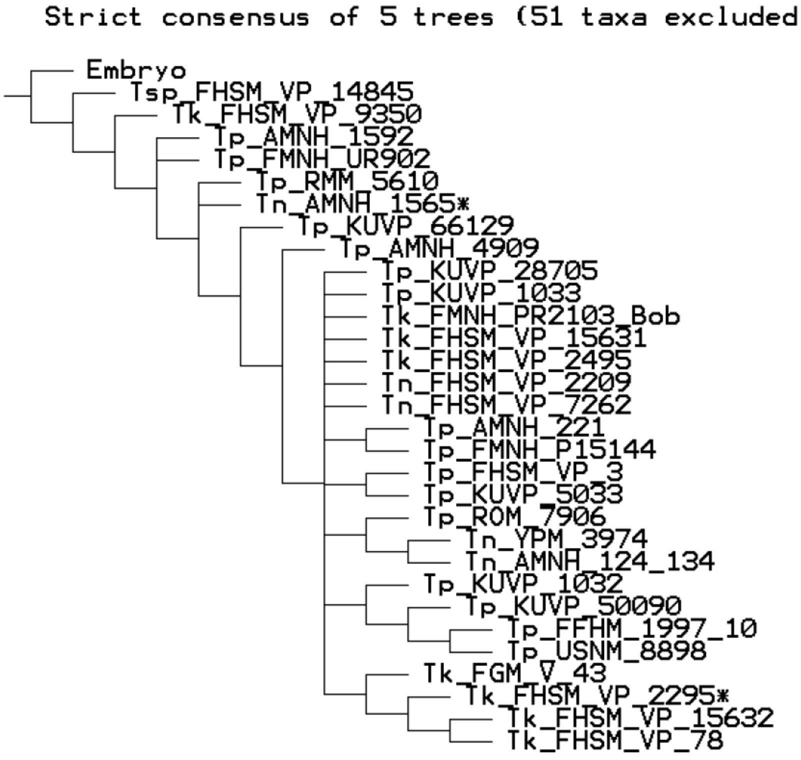

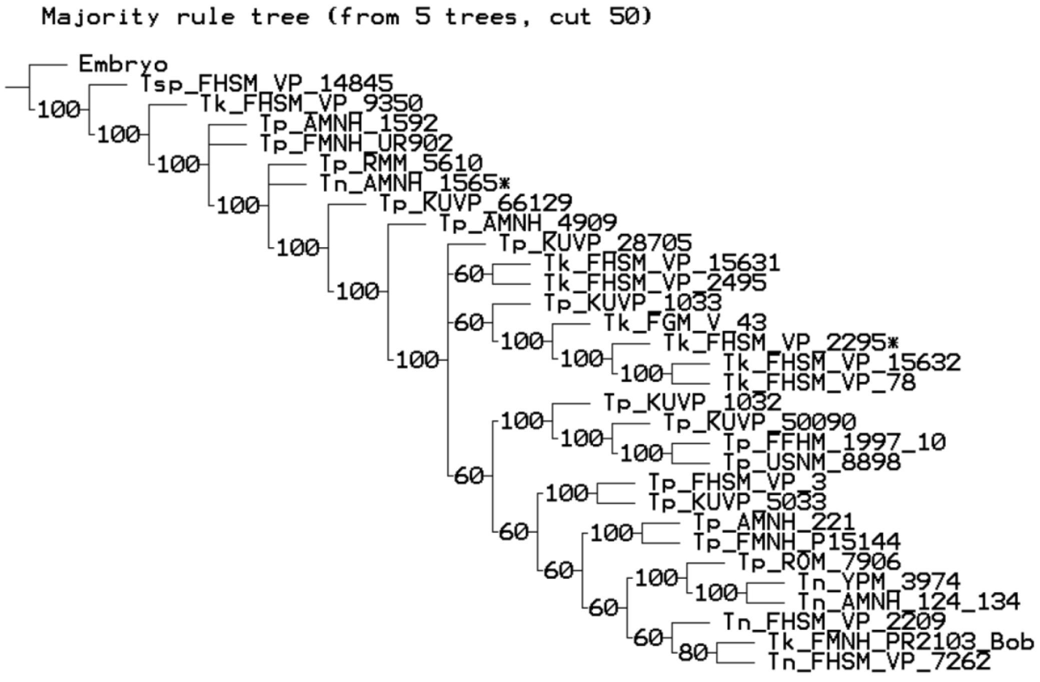

Test L: Strict (top) and 50% majority rule (bottom) ontograms including all specimens of *Tylosaurus proriger* that are scored for five or more characters. Note: an analysis including all specimens resulted in a complete polytomy (not figured).

Strict consensus of 41 trees (14 taxa excluded)

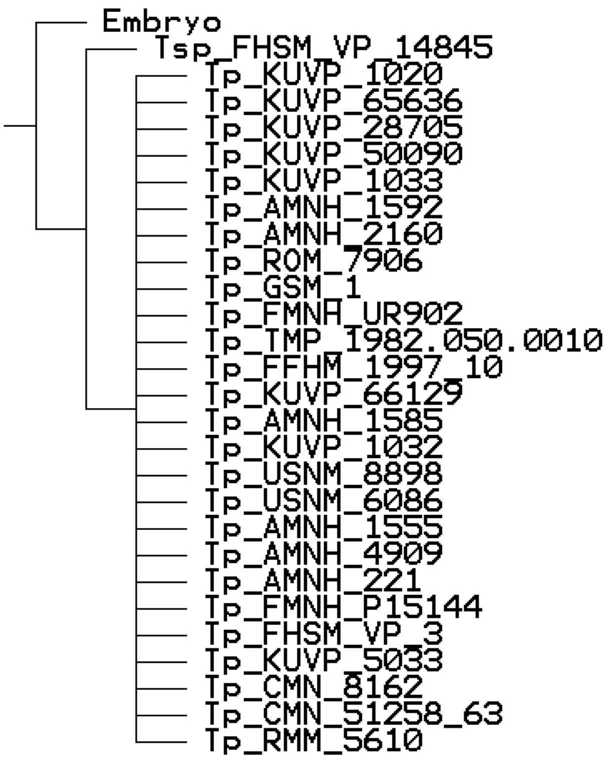

Majority rule tree (from 41 trees, cut 50)

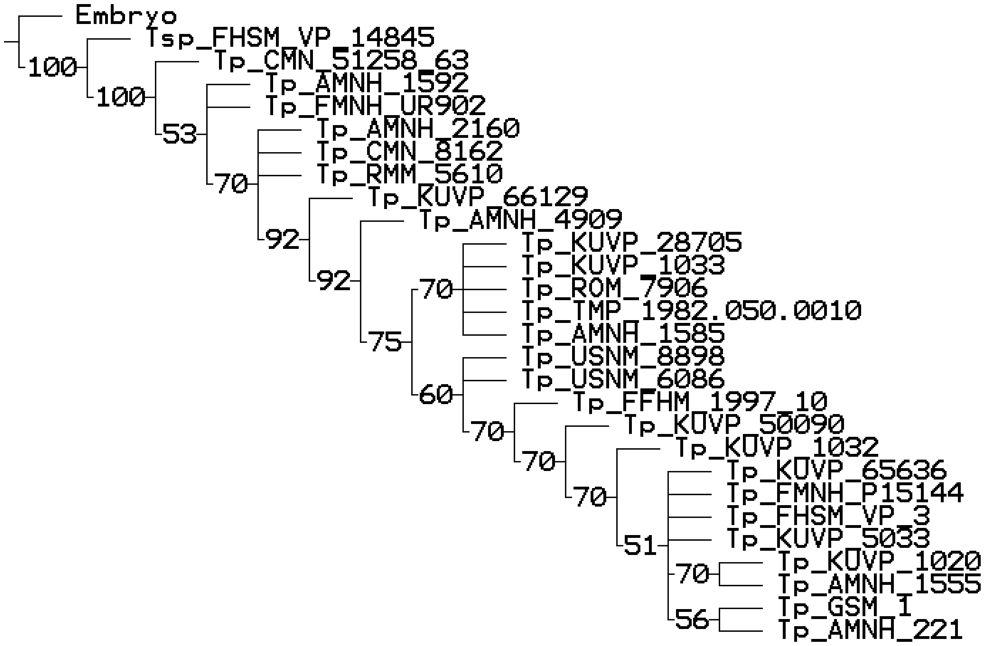

Test M: Strict (top) and 50% majority rule (bottom) ontograms including all specimens of *Tylosaurus kansasensis/nepaeolicus*. Note: TMM 31051-64 is mislabeled in the diagram as TMM 81051-64.

Strict consensus of 38 trees (6 taxa excluded)

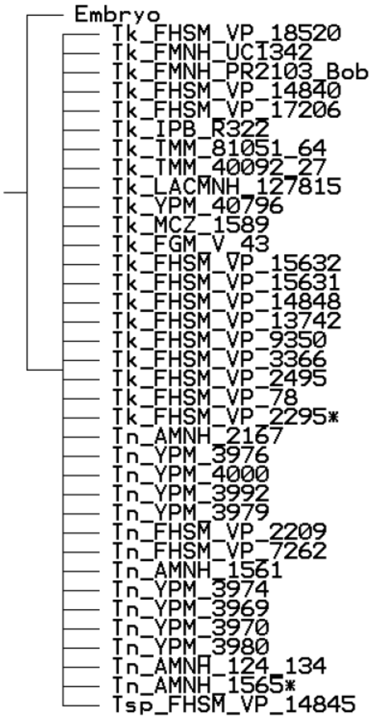

Majority rule tree (from 38 trees, cut 50)

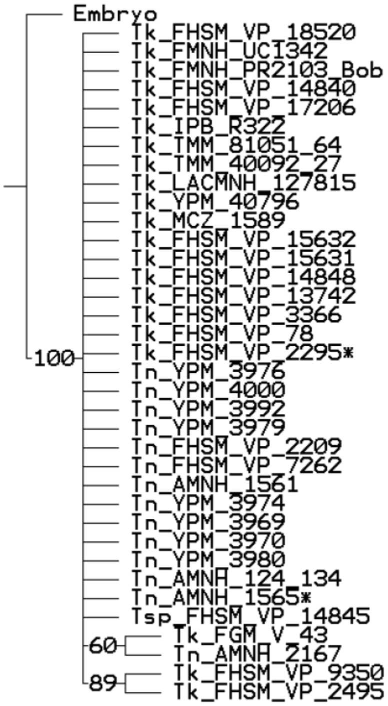

Test N: Strict (top) and 50% majority rule (bottom) ontograms including all specimens of *Tylosaurus kansasensis/nepaeolicus* that are scored for five or more characters.

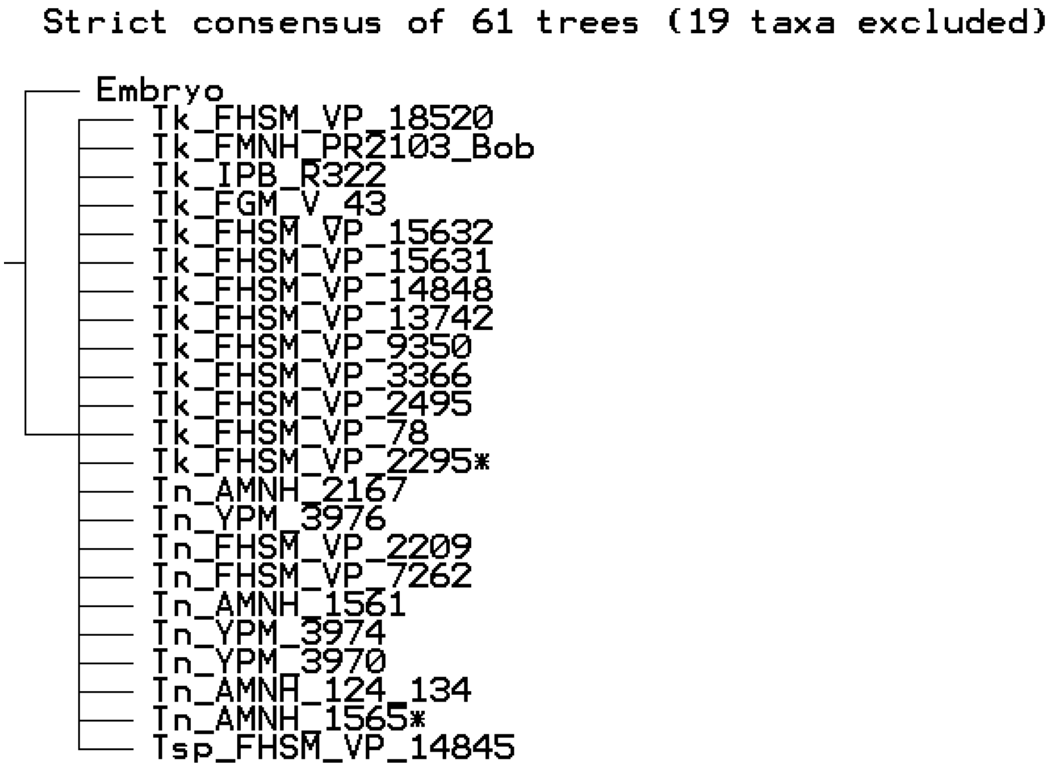

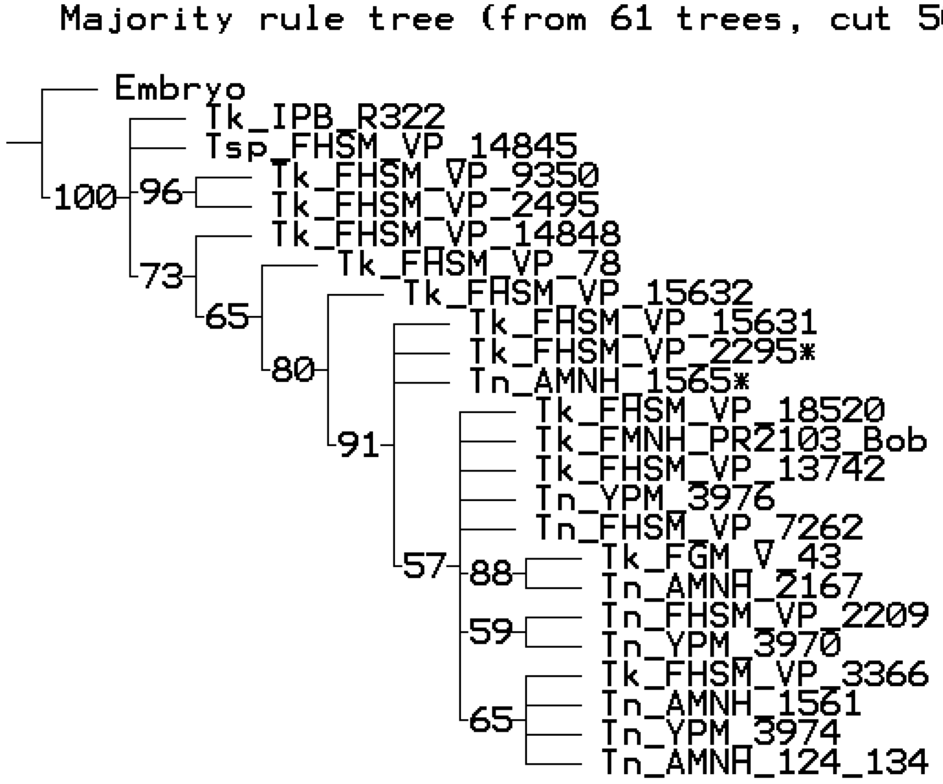

Test O: Strict (left) and 50% majority rule (right) ontograms including all specimens of all three taxa that are scored for five or more characters. Note: an analysis including all specimens resulted in a complete polytomy (not figured). Note: TMM 31051-64 is mislabeled in the diagram as TMM 81051-64.

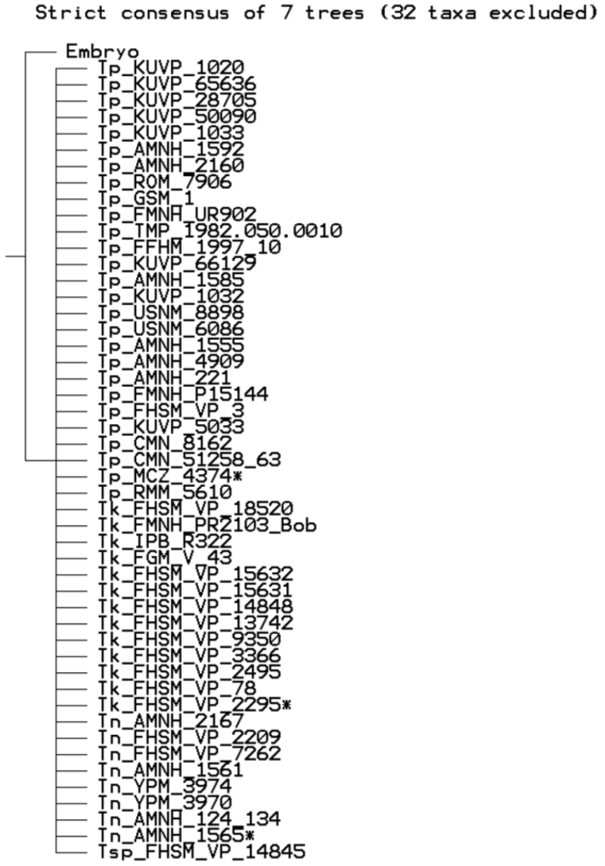

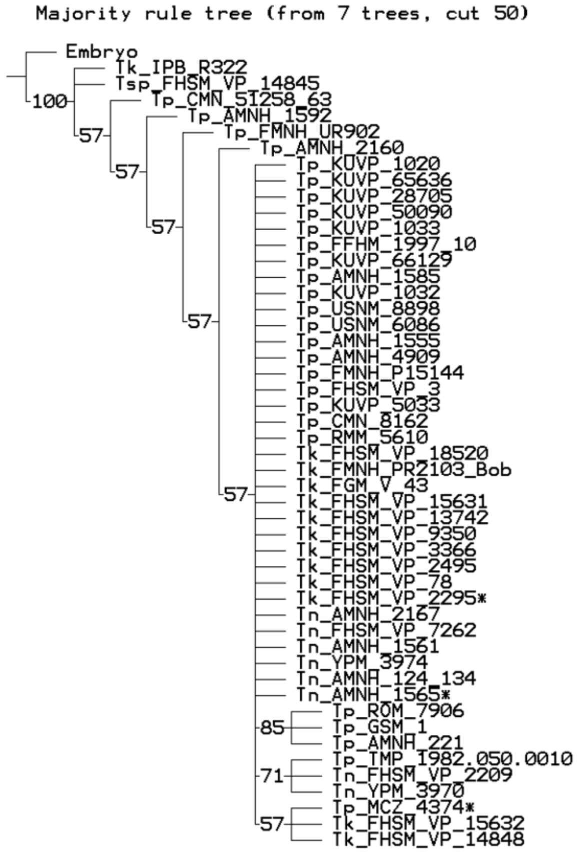

Supplement: Supplemental Information 6 [file peerj-08-10145-s006.pdf]
