# Supplementary material for: Craniofacial ontogeny in Tylosaurinae
Source: PeerJ. 2020 Oct 20;8:e10145. doi: 10.7717/peerj.10145 (PMC7583613; doi:10.7717/peerj.10145)
Supplement: Supplemental Information 7 — A list of all characters is provided in Data S3. Photographs and drawings are not to scale. [file peerj-08-10145-s007.pdf]

Character number 4, premaxilla predental rostrum shape, lateral view. (A) State 0, acute (*T. proriger* AMNH FARB 4909). (B) State 1, rounded and, in *Tylosaurus*, distinct knob present (*T. proriger* FMNH P15144). Note: the photograph of FMNH P15144 is inverted to face left.

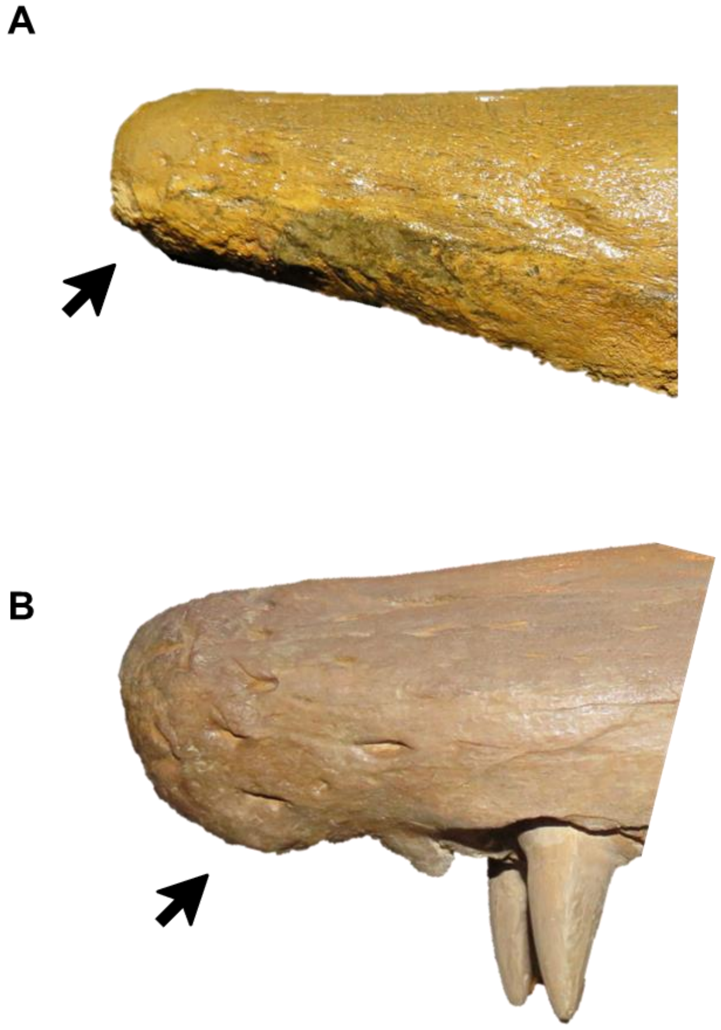

Examples of character number 5, premaxilla predental rostrum foramina size. State 0, large (A, *T. proriger* FHSM VP-2160; D, FHSM VP-14840); state 1, small and pinhole-like (B, KUVP 66129; E, *T. nepaeolicus* FHSM VP-7262); and state 2, both small and large (C, FHSM VP-3). State 0 is slightly different between *T. proriger* (A-C) and *T. nepaeolicus* (D, E): in *T. proriger* the foramina are shallow and oblong, whereas in *T. nepaeolicus* they are deep and circular. State 2, where both small and large foramina are present, is only seen in *T. proriger*. Note: the photograph of FHSM VP-14840 is inverted to face left.

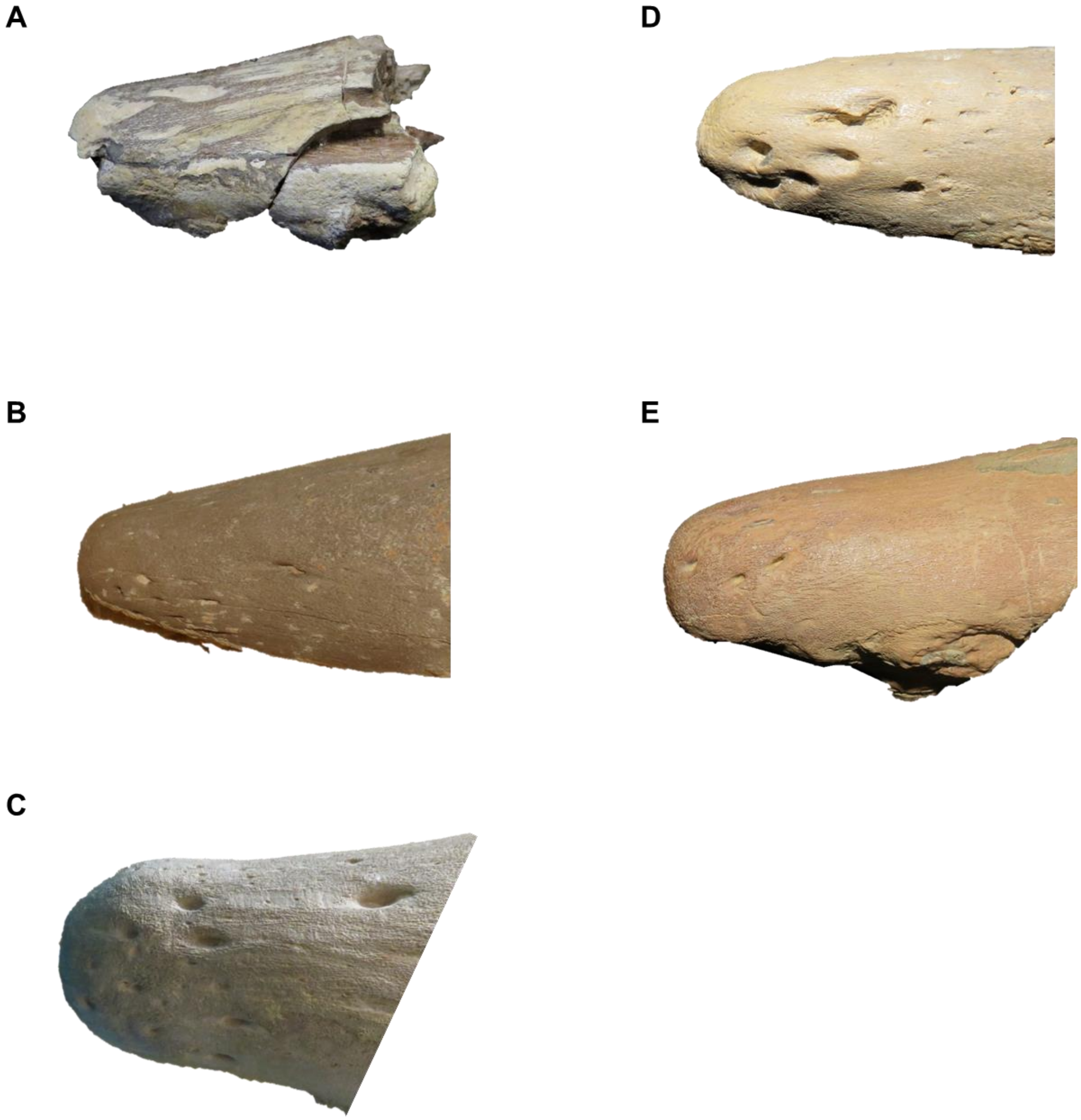

Examples of character number 7, premaxilla-maxilla suture shape. (A) State 0, rectangular (*T. proriger* AMNH FARB 4909). (B) State 1, u-shaped (*T. proriger* FHSM VP-3). (C) State 2, m-shaped (*T. proriger* FMNH P15144). Note: the photograph of AMNH FARB 4909 is inverted to face left.

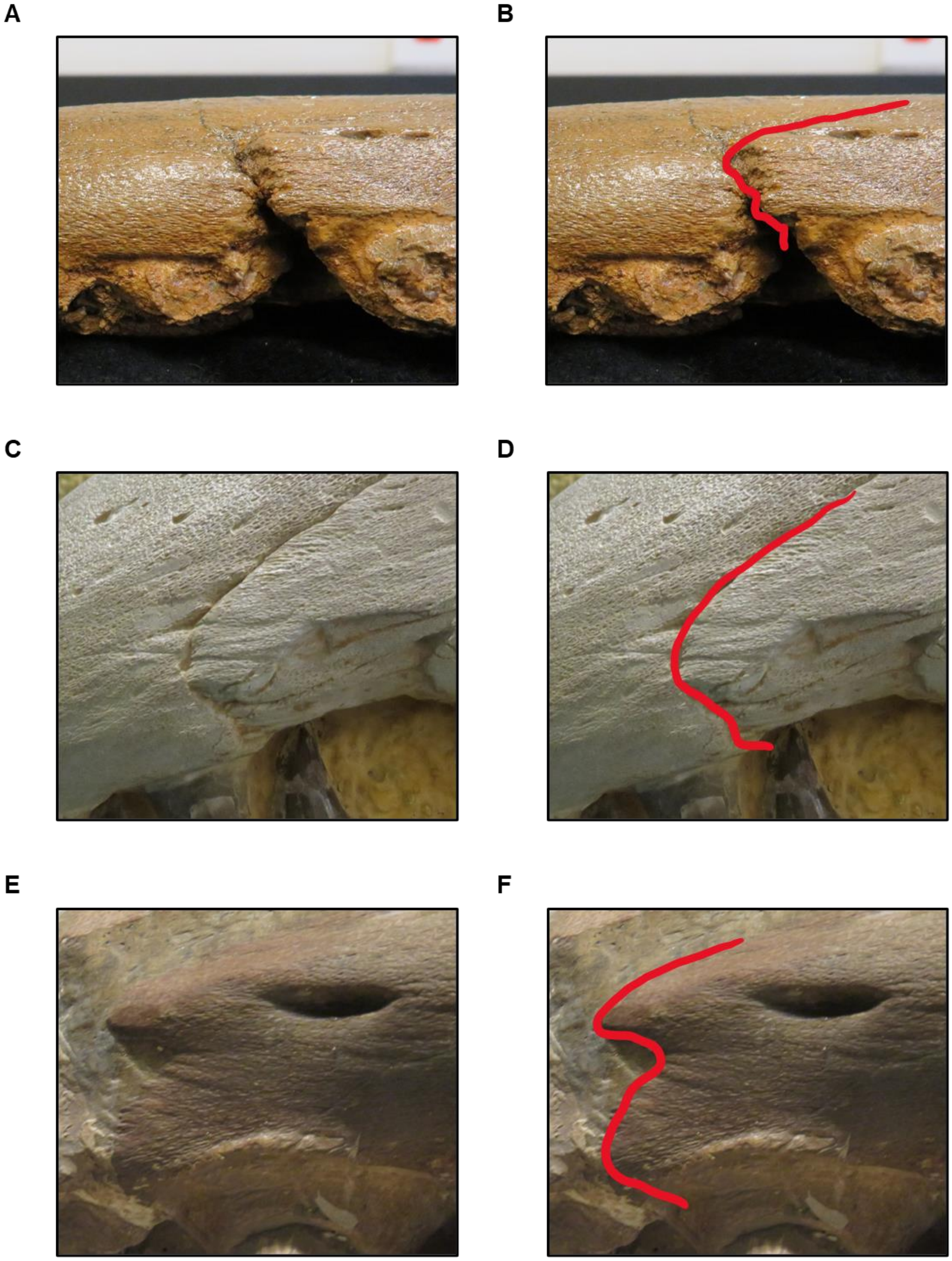

Examples of character number 5, quadrate suprastapedial process length, lateral view. State 0, short (A, *T. proriger* FHSM VP-2160; C, *T. nepaeolicus* FHSM VP-9350); state 1, reaches midheight (B, KUVP 66129; D, FHSM VP-14840); and state 2, long (E, *T. nepaeolicus* FHSM VP-7262); the only major difference between *T. proriger* (A, B) and *T. nepaeolicus* (C-E) is that no *T. proriger* specimens have a long suprastapedial process (state 2). Red dashed lines indicate approximately mid-height of the bone. Note: the photographs of FHSM VP-9350 and AMNH FARB 4909 are left quadrates that have been inverted.

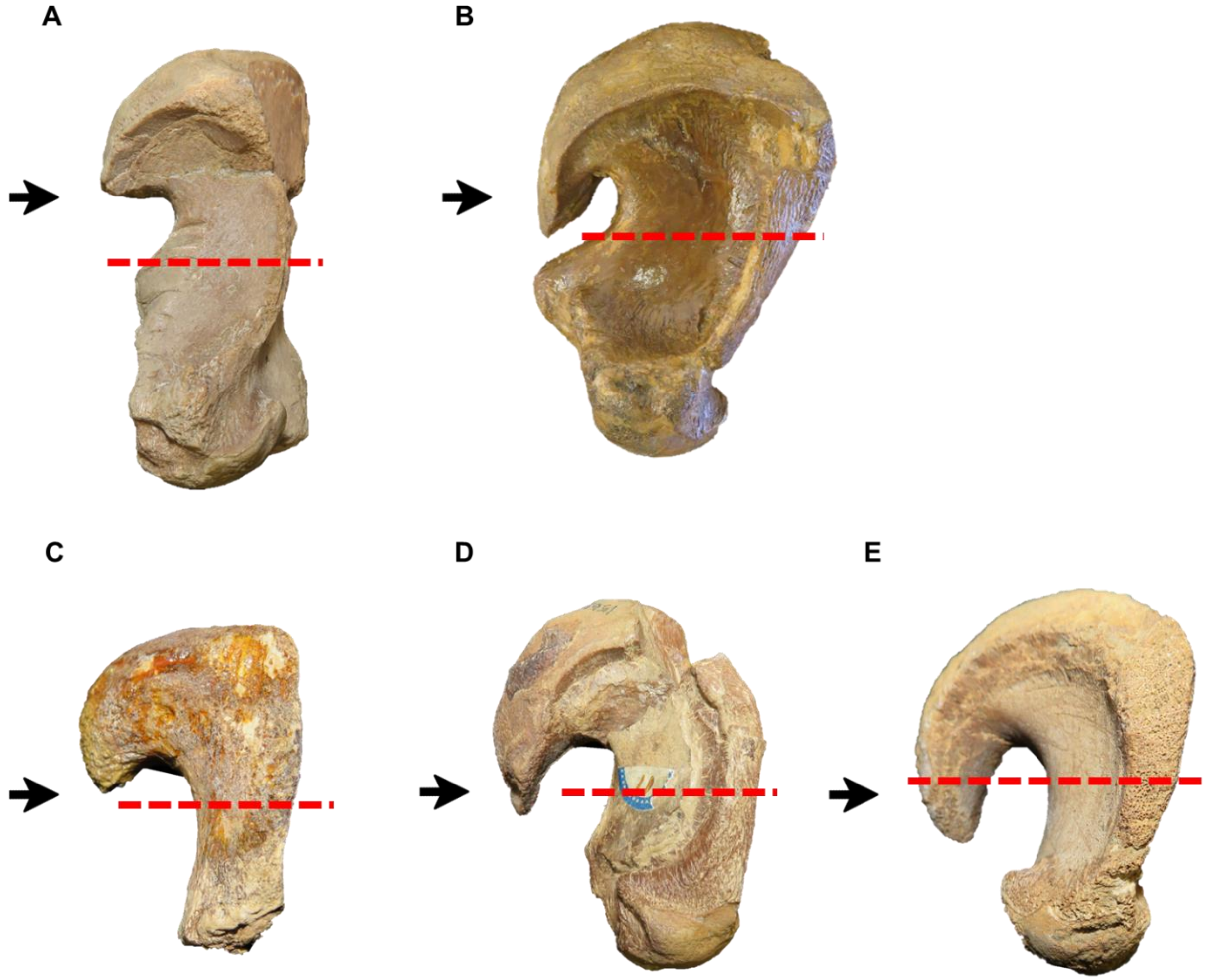

Examples of character number 12, quadrate suprastapedial process thickness, lateral view. State 0, slender (A, AMNH FARB 4909; C, AMNH FARB 1565); and state 1, robust (B, AMNH FARB 1555; D, FGM V-43); the main difference between *T. proriger* (A, B) and *T. nepaeolicus* (C, D) is that state 1 is more pronounced in *T. nepaeolicus*. Note: the photographs of AMNH FARB 4909 and AMNH FARB 1555 are left quadrates that have been inverted.

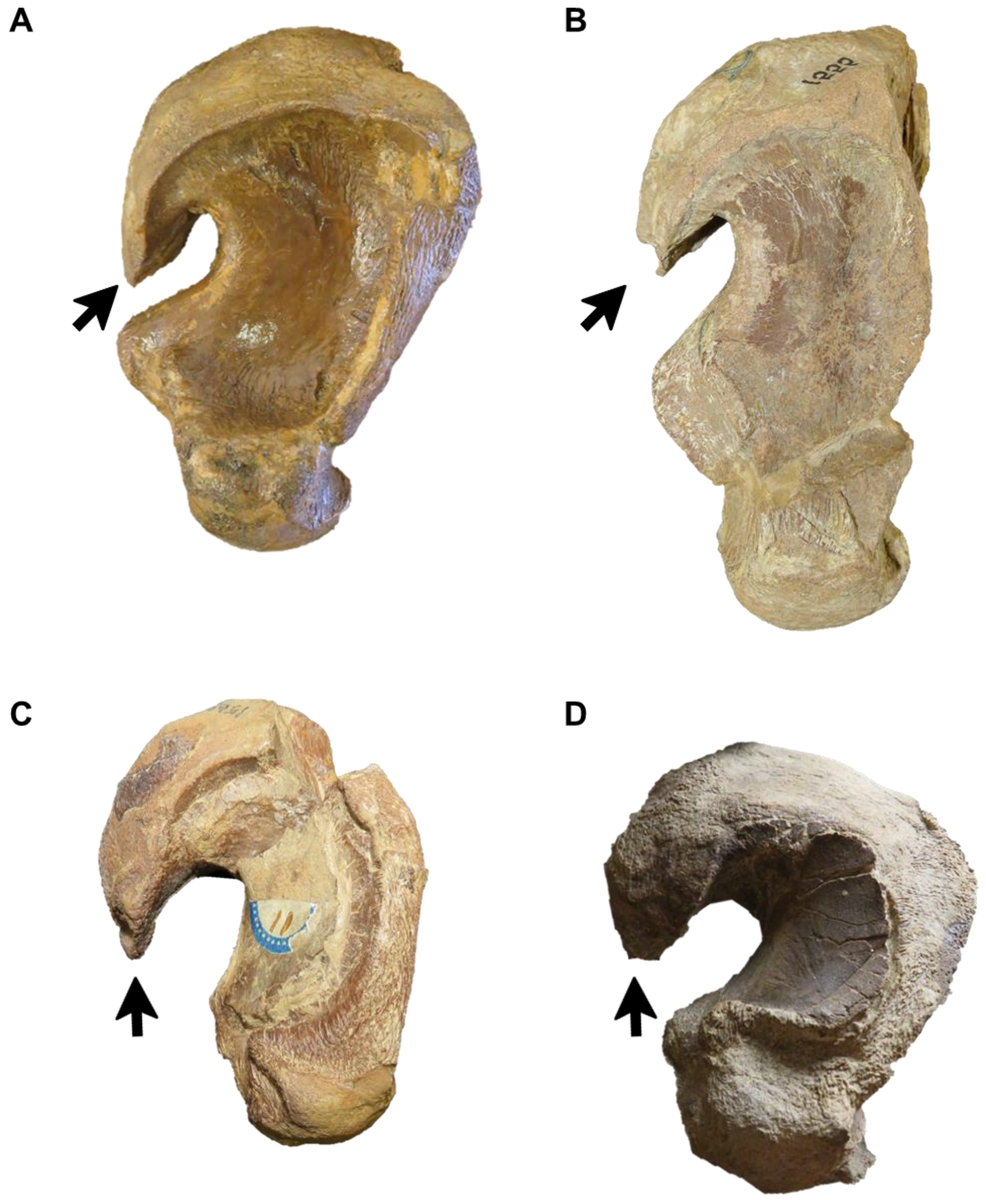

Examples of character number 13, medial curvature of the quadrate suprastapedial process, posterior view. (A) State 0, present (*T. nepaeolicus* FHSM VP-9350). (B) State 1, absent (*T. nepaeolicus* AMNH FARB 2167). Red dashed lines indicate the curvature of the suprastapedial process relative to the main body of the quadrate. Note: FHSM VP-9350 was previously identified as *T. kansasensis*.

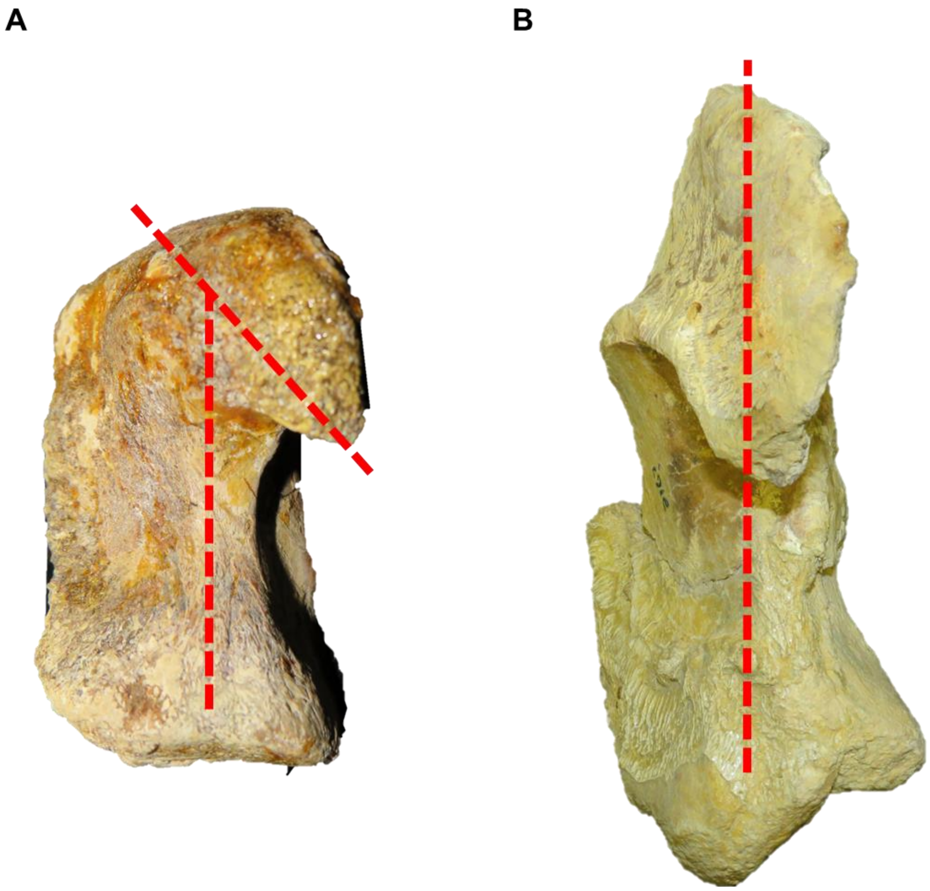

Examples of character numbers 15 and 16, quadrate infrastapedial process development and shape, lateral view. In *T. proriger*, (A, FMNH UR902; B, AMNH FARB 1555), the infrastapedial process is always present (character number 15, state 1) and can be either broadly pointed (A; character number 16, state 0) or expanded, rounded, and semicircular (B; character number 16, state 1). In *T. nepaeolicus* (A, FHSM VP-15632; B, AMNH FARB 1565; C, FGM V-43), the infrastapedial process can be absent (C, character number 15, state 0), present and broadly pointed (B, character number 15, state 1 and character number 16, state 0), or present and expanded, rounded, and semicircular (C, character number 15, state 1 and character number 16, state 1). Notes: the photograph of AMNH FARB 1555 is the left quadrate that has been inverted; FHSM VP-15632 and FGM V-43 were previously identified as *T. kansasensis*.

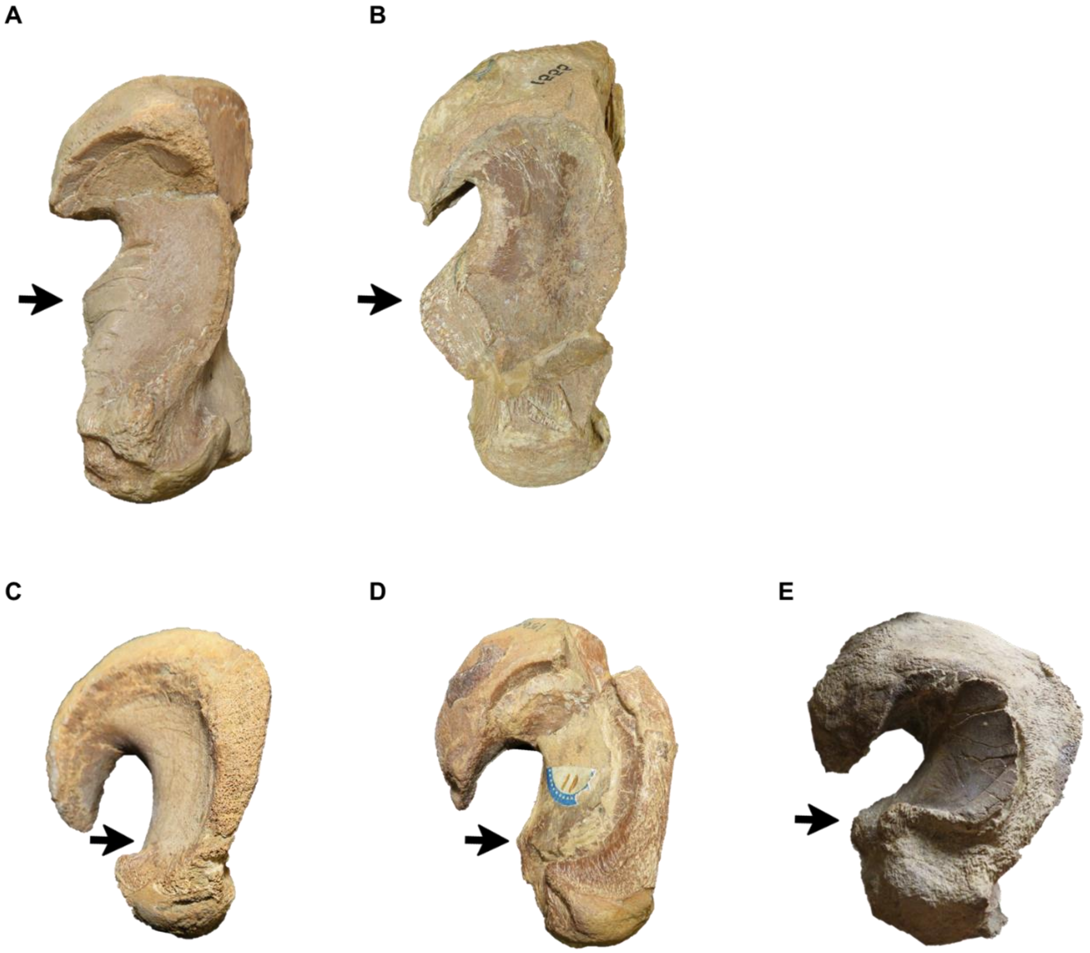

Examples of character number 20, anteroventral corner of the quadrate tympanic ala, lateral view. (A) State 0, absent (based on *Mosasaurus hoffmannii* TMM 313-1 (Harrell & Martin, 2015)). (B) State 1, present (based on *Mosasaurus hoffmannii* MNHN AC 9648 (Harrell & Martin, 2015)).

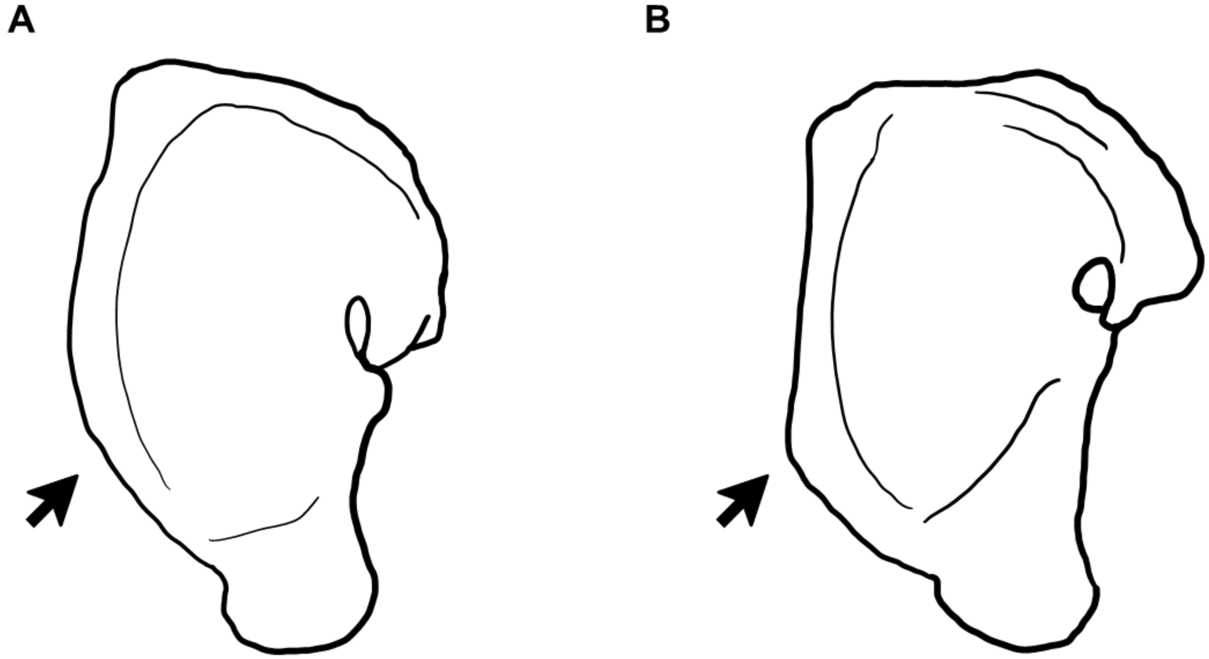

Examples of character number 26, anterodorsal deflection of the quadrate mandibular condyle, lateral view. (A) State 0, present (*T. proriger* FMNH UR902). (B) State 1, absent, condyle rounded (*T. proriger* AMNH FARB 1555). Note: the photograph of AMNH FARB 1555 is the left quadrate that has been inverted.

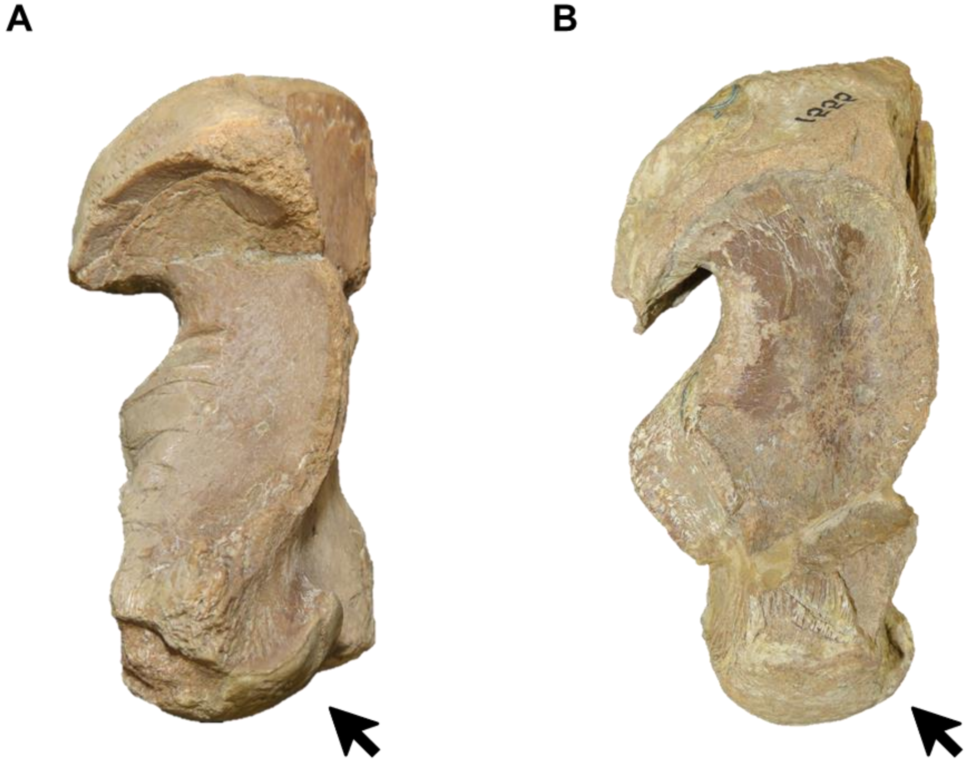

Examples of character number 28, frontal posterolateral process shape. (A) State 0, slender (*T. proriger* KUVP 28705). State 1, robust (*T. proriger* KUVP 65636).

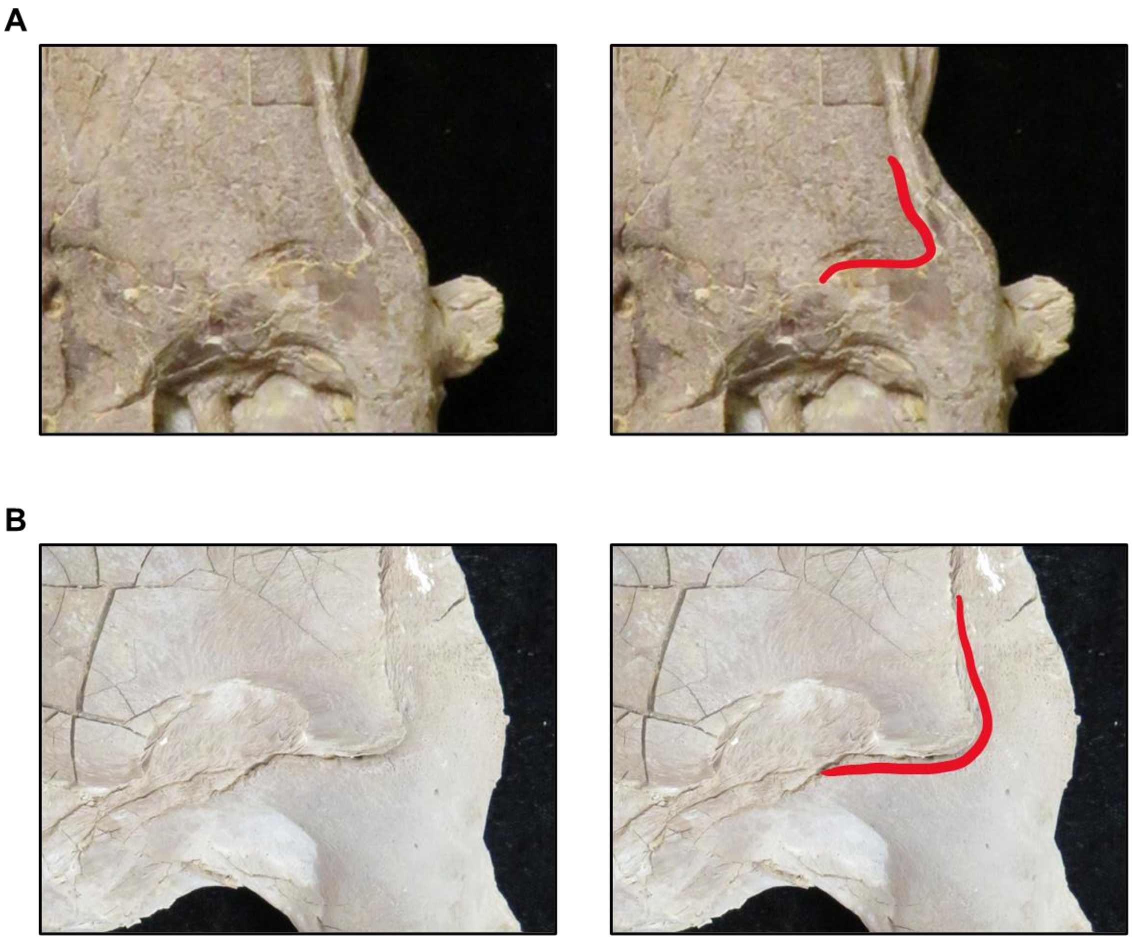

Examples of character number 32, frontal-parietal medial suture flange size. State 0, large (**A**, *T. proriger* KUVP 28705; **C**, *T. nepaeolicus* FHSM VP-2295); state 1, small (**B**, *T. proriger* AMNH FARB 4909; **D**, *T. nepaeolicus* FHSM VP-15631). The main difference between *T. proriger* and *T. nepaeolicus* is that state 0 is more pronounced in *T. nepaeolicus*. Note: FHSM VP-2295 and FHSM VP-15631 were previously identified as *T. kansasensis*.

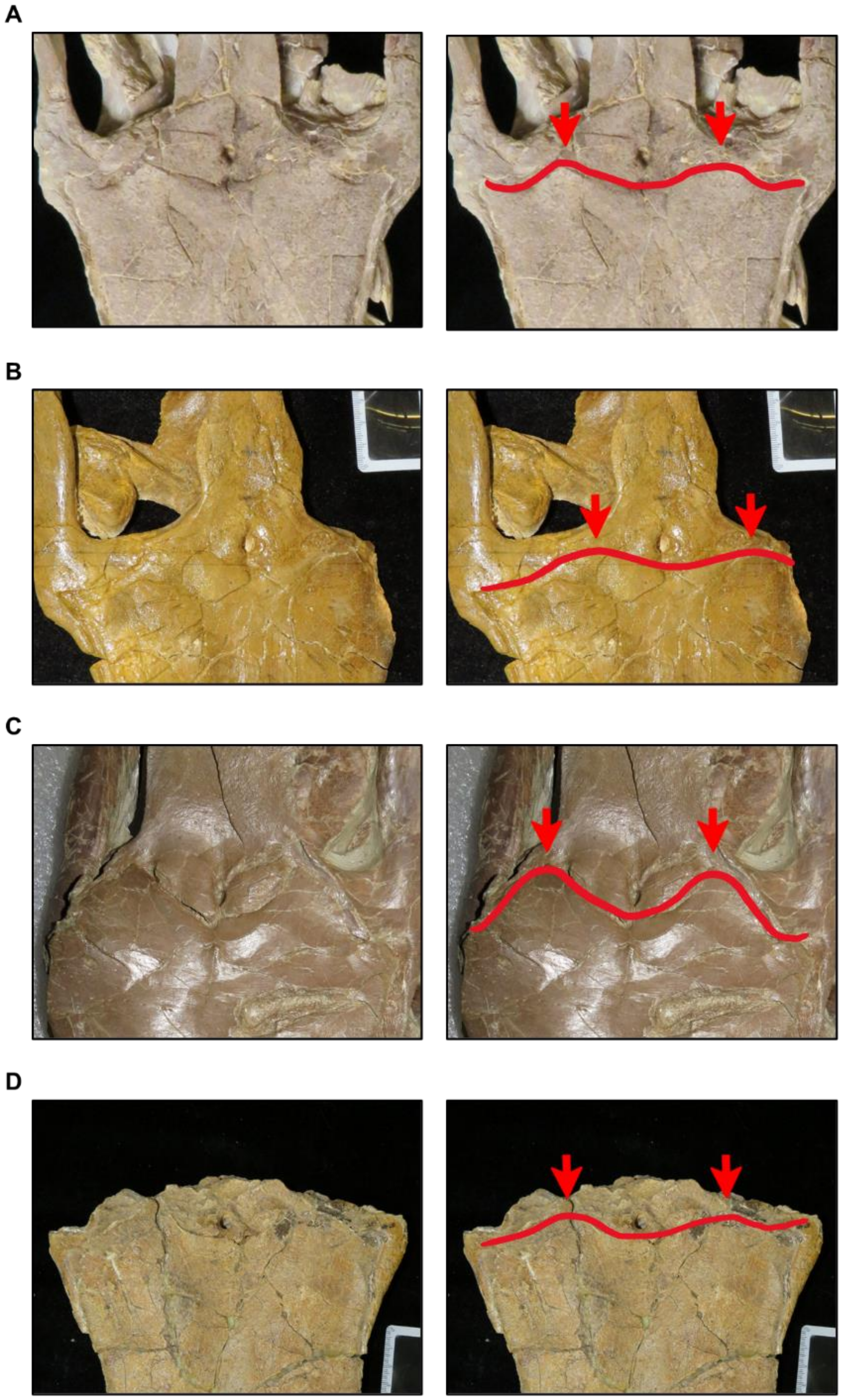

Examples of character number 35, parietal posterior pegs, dorsal view. (A) State 0, absent (*T. nepaeolicus* FHSM VP-2209). (B) State 1, present and small (*T. nepaeolicus* FHSM VP-2295). (C) State 2, present and large (based on *Tethysaurus nopcsai* MNHN GOU 2 (Bardet, Suberbiola, & Jalil, 2003)). Note: FHSM VP-2295 was previously identified as *T. kansasensis*.

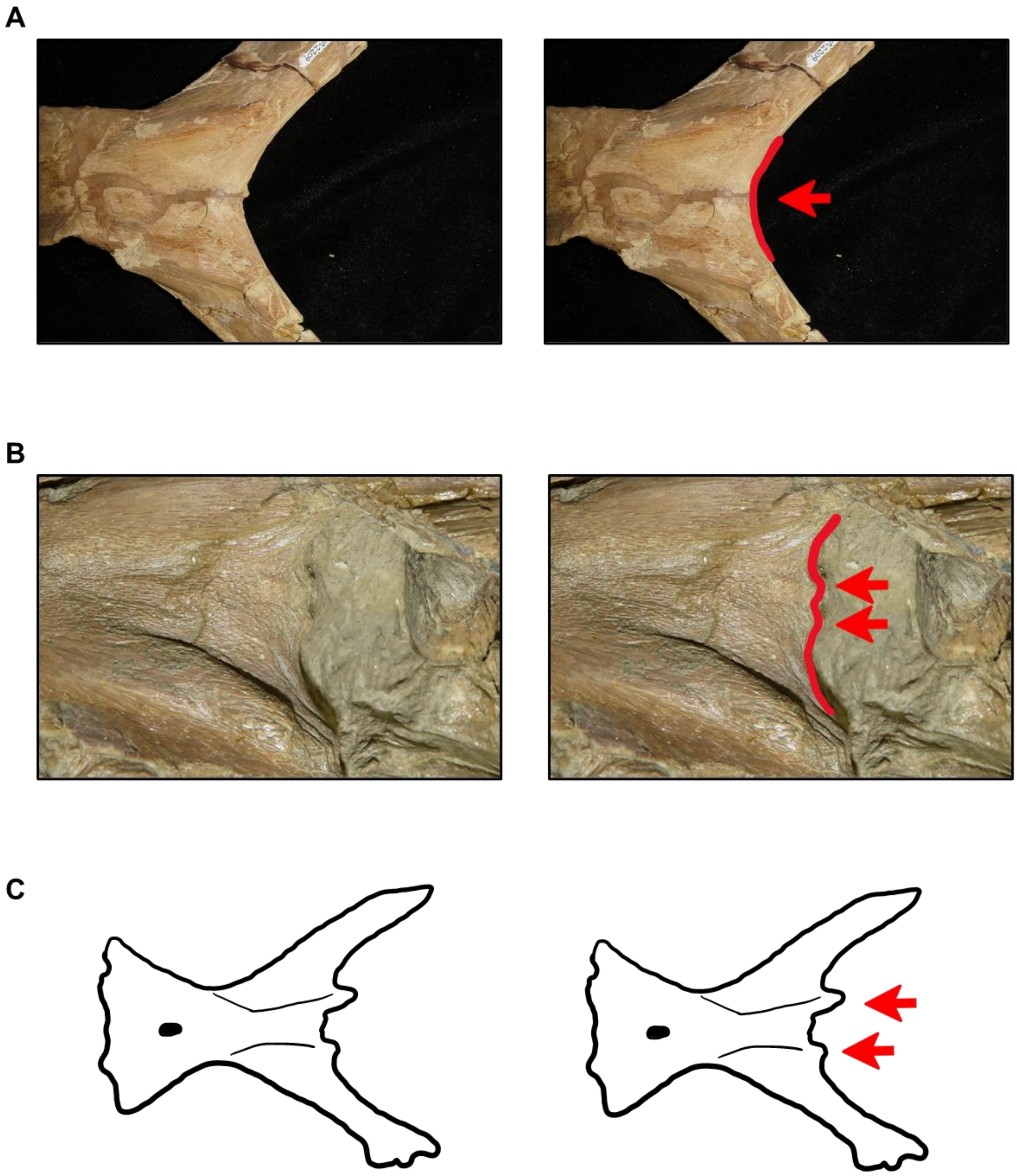

Examples of character number 37, parietal nuchal fossa, dorsal view. (A) State 0, posterodorsal surface of parietal flat (*T. proriger* FMNH P15144). (B) State 1, fossa present (*T. proriger* AMNH FARB 4909).

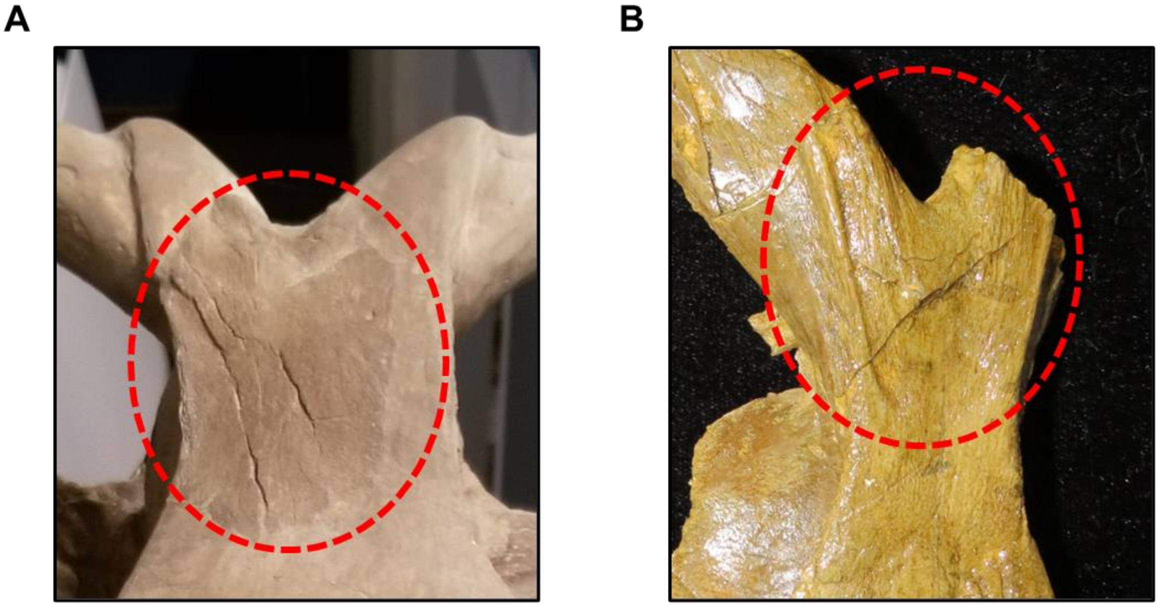

Examples of character number 38, parietal lateral border shape, dorsal view. (A) State 0, convex (*T. nepaeolicus* FHSM VP-78). (B) State 1, straight (*T. nepaeolicus* FHSM VP-2209). Note: FHSM VP-78 was previously identified as *T. kansasensis*.

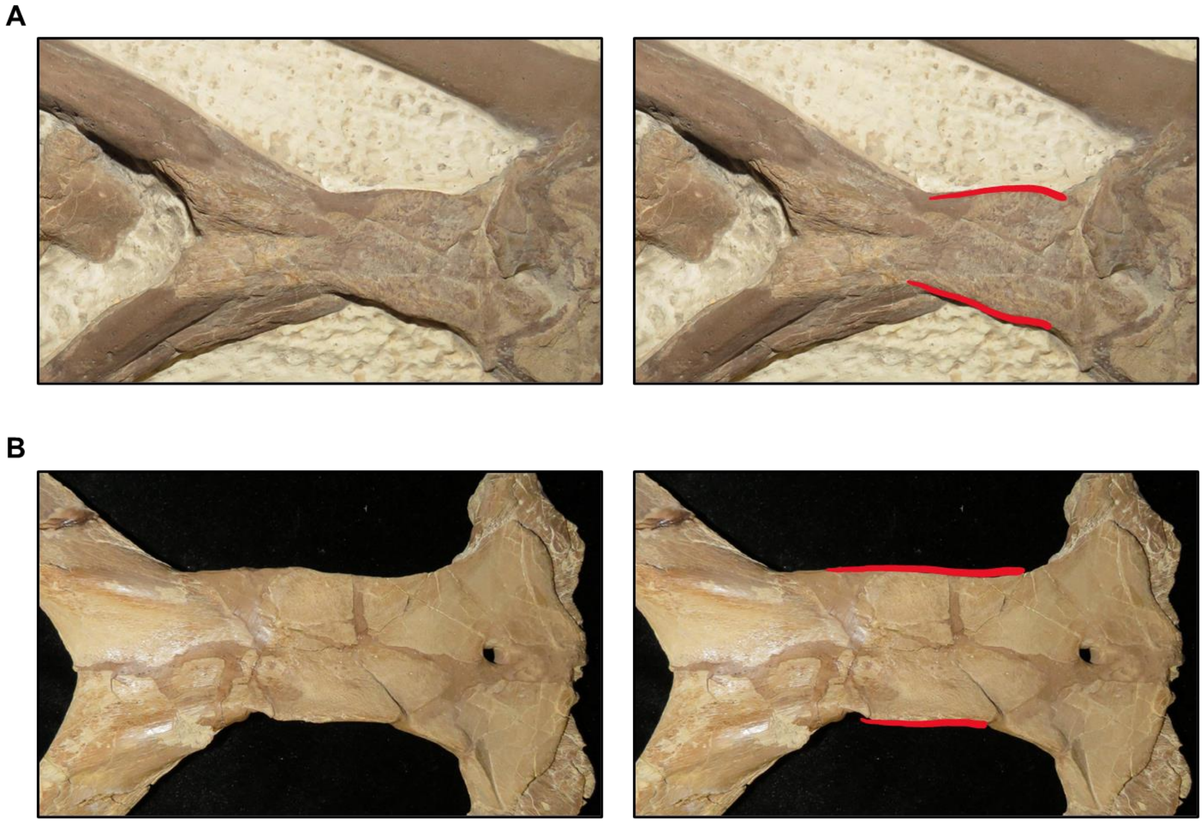

Examples of character number 40, jugal posteroventral process, lateral view. (A) State 0, absent (*T. proriger* AMNH FARB 4909). (B) State 1, present (*T. proriger* FHSM VP-3). Note: the photograph of AMNH FARB 4909 is the right jugal that has been inverted.

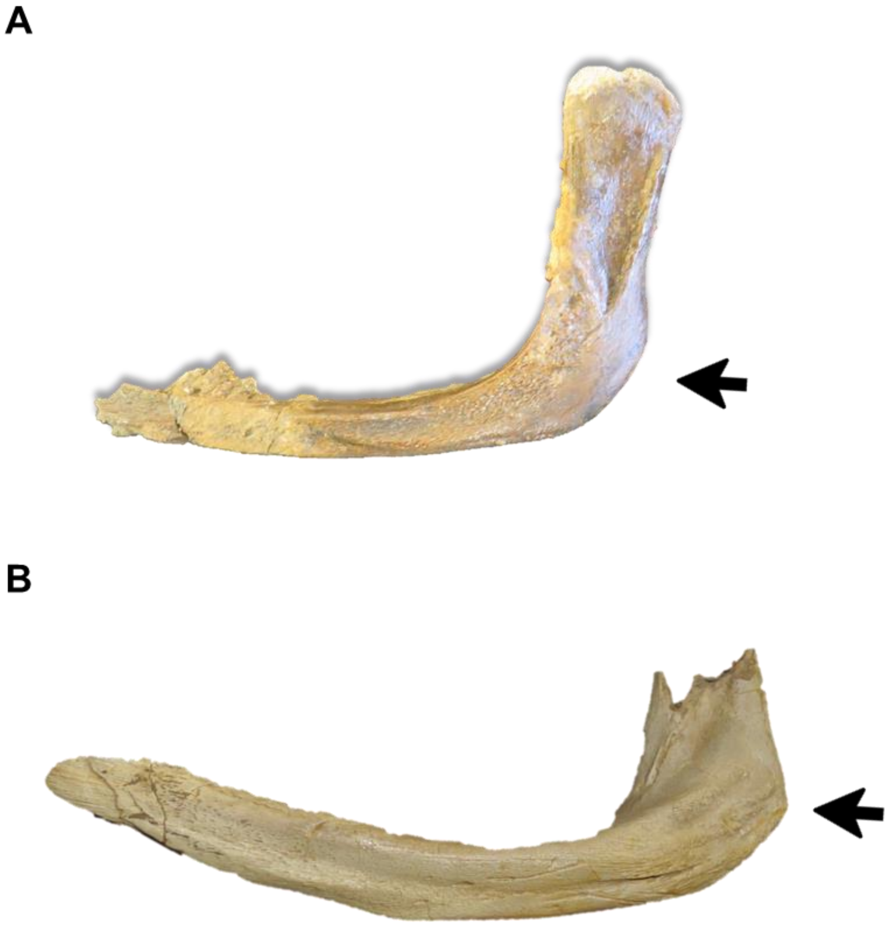

Examples of character number 45, ectopterygoid process shape, dorsal and ventral (figured here) views. (A) State 0, slender (*T. proriger* AMNH FARB 4909). State 1, robust (*T. proriger* FHSM VP-3).

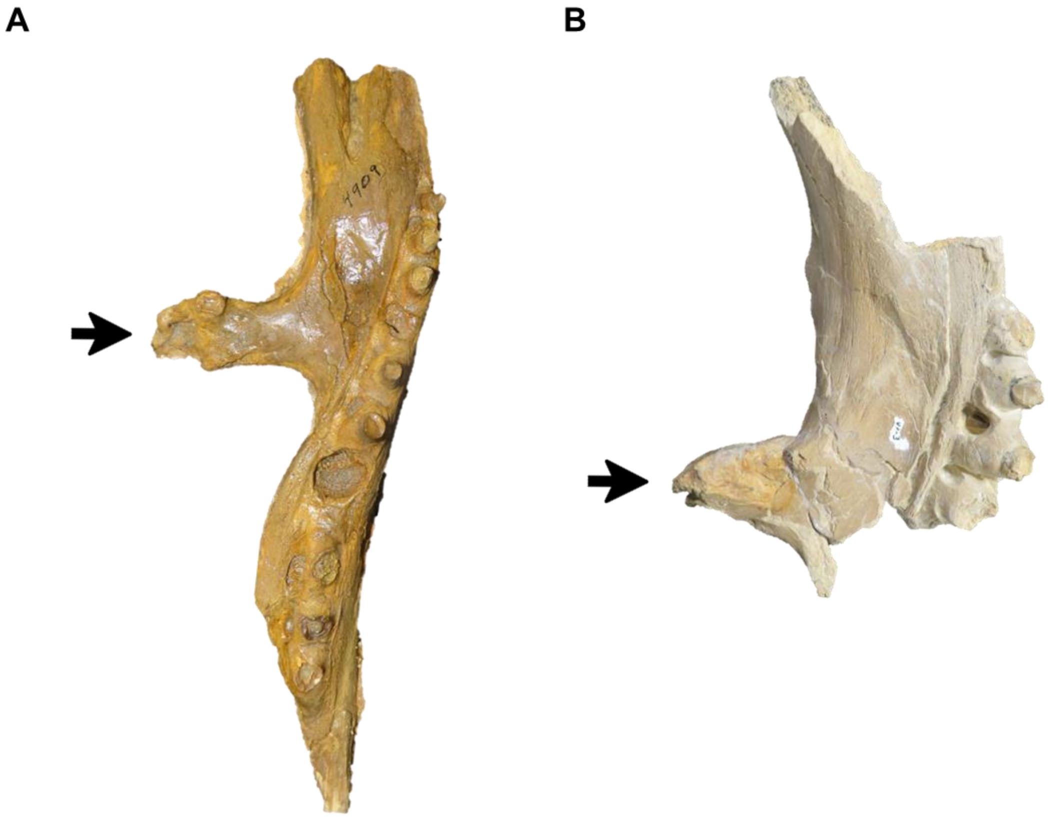

Examples of character number 50, dorsal ridge of dentary predental process, lateral view. (A) State 0, absent (*T. proriger* AMNH FARB 4909). (B) State 1, present (*T. proriger* FMNH UR820).

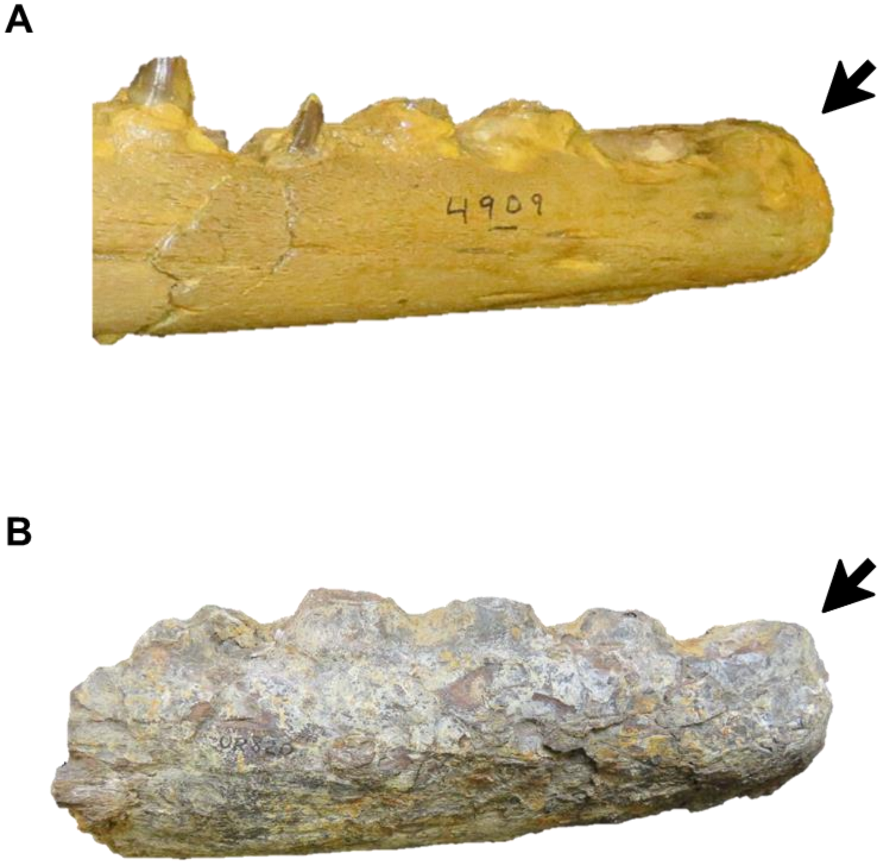

Examples of character number 57, coronoid anterolateral flange notch, lateral view. (A) State 0, absent (*T. nepaeolicus* AMNH FARB 1565). (B, C) State 1, notch present and shallow (B, *T. nepaeolicus* FHSM VP-2209; C, based on *Mosasaurus hoffmannii* TMM 11202 (Harrell & Martin, 2015)). (D) State 2, notch present and deeply c-shaped (based on *Mosasaurus hoffmannii* IRSNB R 27 (Harrell & Martin, 2015)).

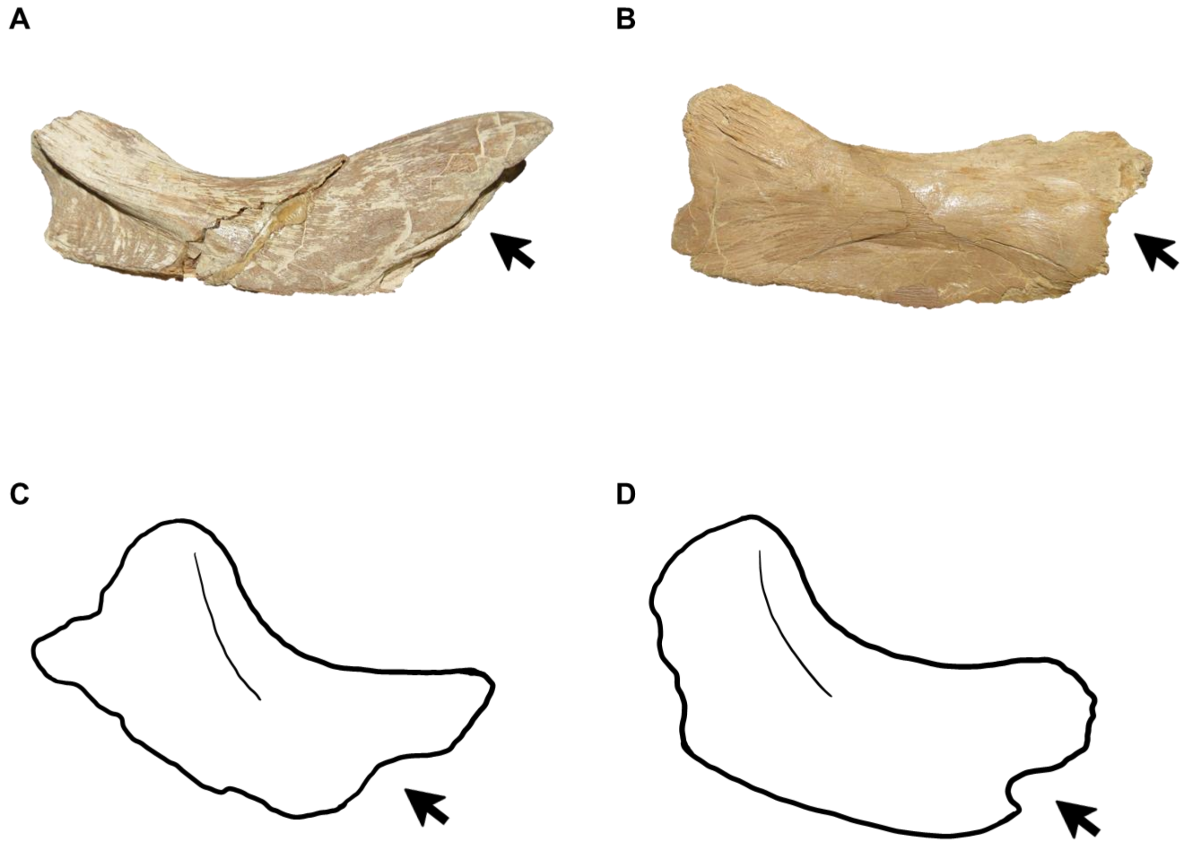

Examples of character number 58, coronoid posteroventral process, lateral view. (A) State 0, absent (*Tylosaurus* sp. FHSM VP-14845). (B) State 1, process present as bump (*T. proriger* FHSM VP-3). (C) State 2, process present and fan-like (*T. proriger*, KUVP 5033). Note: the photograph of KUVP 5033 is a right coronoid that has been inverted.

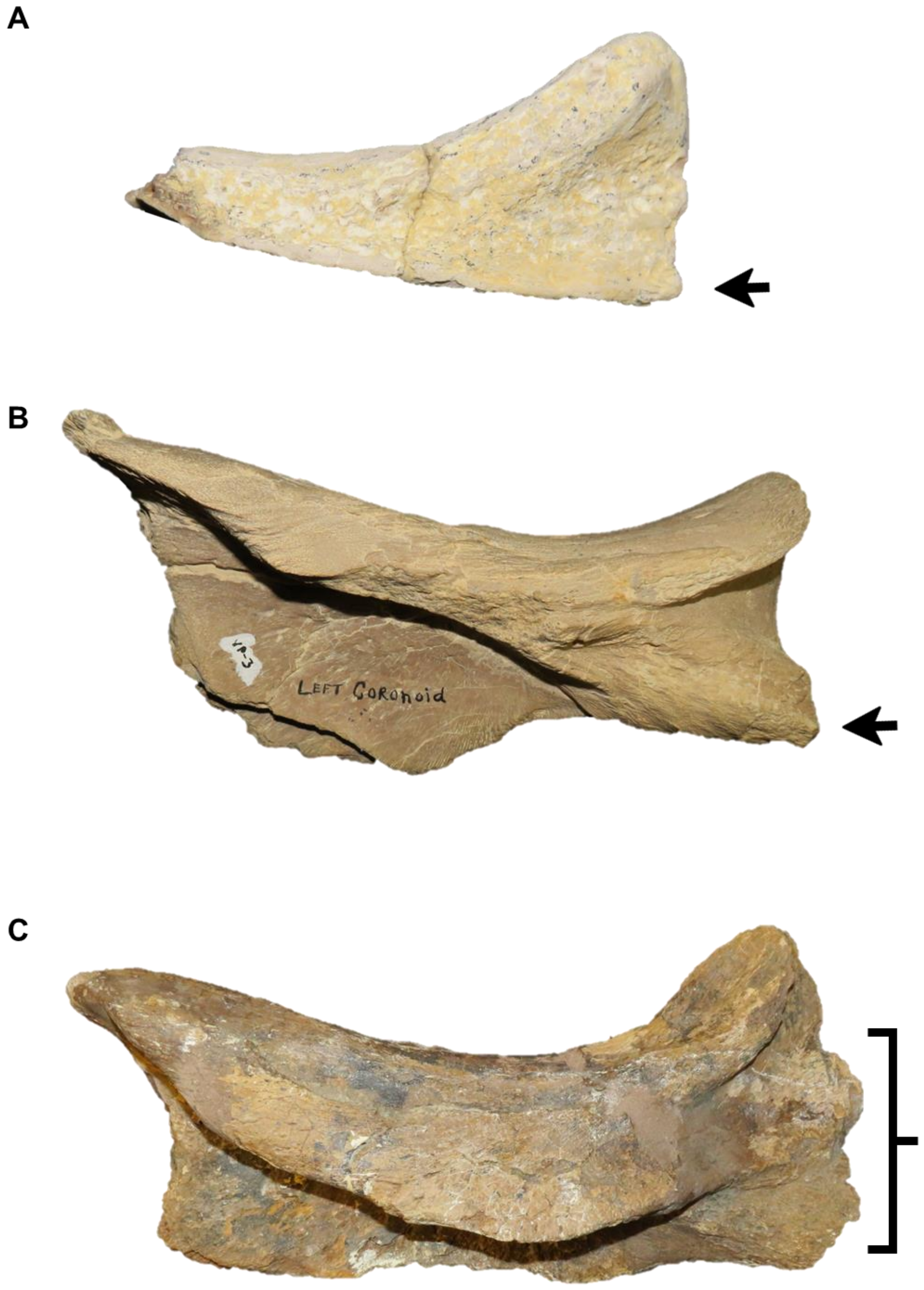

References

Bardet N, Suberbiola XP, Jalil NE. 2003. A new mosasauroid (Squamata) from the Late Cretaceous (Turonian) of Morocco. Comptes Rendus Palevol 2:607-616.

Harrell TL, Martin JE. 2015. A mosasaur from the Maastrichtian Fox Hills Formation of the northern Western Interior Seaway of the United States and the synonymy of *Mosasaurus maximus* with *Mosasaurus hoffmanni* (Reptilia: Mosasauridae). Netherlands Journal of Geosciences 94:23-37.
