# Supplementary material for: Craniofacial ontogeny in Tylosaurinae
Source: PeerJ. 2020 Oct 20;8:e10145. doi: 10.7717/peerj.10145 (PMC7583613; doi:10.7717/peerj.10145)
Supplement: Supplemental Information 8 — Type specimens are indicated by an asterisk. TMP 1982.050.0010 is a cast of LACMNH 28964; CMN 51258 through 51263 are fragments from a single individual (Stewart & Mallon, 2018); AMNH FARB 124 and 134 are a skull and jaws, respectively, from a single individual (Jiménez-Huidobro & Caldwell, 2019). [file peerj-08-10145-s008.docx]

| Specimen | Taxon | References for Coding | Measurement Sources |
| --- | --- | --- | --- |
| RMM 5610 | *T. proriger* | Jiménez-Huidobro, Simões, & Caldwell, 2016; Konishi, Jiménez-Huidobro, & Caldwell, 2018 | Jiménez-Huidobro, Simões, & Caldwell, 2016 and Konishi, Jiménez-Huidobro, & Caldwell, 2018; estimates given and scale bars |
| *MCZ 4372 | *T. proriger* | Oceans of Kansas website (Everhart, n.d.); Cope, 1869 (Pl. XII) | None found |
| KUVP 1129 | *T. proriger* | Jiménez-Huidobro, Simões, & Caldwell, 2016 | None found |
| AMNH FARB 1543 | *T. proriger* | Jiménez-Huidobro, Simões, & Caldwell, 2016 | None found |
| CMN 51258-51263 | *T. proriger* | Stewart & Mallon, 2018 | Stewart & Mallon, 2018; scale bar |
| CMN 8162 | *T. proriger* | Stewart & Mallon, 2018; T. Konishi, 2019, pers. comm. | Stewart & Mallon, 2018;  measurements given |
| KUVP 5033 | *T. proriger* | Personal observation | Personal observation; TSL estimate given by Konishi, Jiménez-Huidobro, & Caldwell, 2018 |
| FHSM VP-3 | *T. proriger* | Personal observation | Personal observation |
| FMNH P15144 | *T. proriger* | Personal observation | Personal observation |
| AMNH FARB 221 | *T. proriger* | Personal observation | Personal observation |
| AMNH FARB 4909 | *T. proriger* | Personal observation | Personal observation |
| AMNH 1555 | *T. proriger* | Personal observation | Personal observation |
| HMG 1288 | *T. proriger* | Oceans of Kansas website (Everhart, n.d.); Stewart & Mallon, 2018 | None found |
| USNM 6086 | *T. proriger* | Oceans of Kansas website (Everhart, n.d.); Russell, 1967 | Russell, 1967; measurements given |
| USNM 8898 | *T. proriger* | Oceans of Kansas website (Everhart, n.d.); Russell, 1967 | Russell, 1967; measurements given |
| YPM 3990 | *T. proriger* | Konishi, Jiménez-Huidobro, & Caldwell, 2018 | None found |
| YPM 1268 | *T. proriger* | Russell, 1967 | Russell, 1967; measurements given |
| YPM 3977 | *T. proriger* | Russell, 1967 | Russell, 1967; measurements given |
| YPM 4002 | *T. proriger* | Russell, 1967 | Russell, 1967; measurements given |
| YPM 3981 | *T. proriger* | Russell, 1967 | Russell, 1967; measurements given |
| KUVP 1032 | *T. proriger* | Personal observation | Personal observation |
| AMNH FARB 1585 | *T. proriger* | Personal observation | Personal observation |
| KUVP 66129 | *T. proriger* | Personal observation | Personal observation |
| FFHM 1997-10 | *T. proriger* | Everhart, 2002; Oceans of Kansas website (Everhart, n.d.) | Everhart, 2002; measurements given |
| TMP 1982.050.0010 | *T. proriger* | Stewart & Mallon, 2018 | Stewart & Mallon, 2018; measurements given |
| FMNH UR902 | *T. proriger* | Personal observation | Personal observation |
| FMNH UR820 | *T. proriger* | Personal observation | Personal observation |
| GSM 1 | *T. proriger* | Stewart & Mallon, 2018 | Stewart & Mallon, 2018; measurements given |
| ROM 7906 | *T. proriger* | Stewart & Mallon, 2018 | Stewart & Mallon, 2018; measurements given |
| AMNH FARB 2160 | *T. proriger* | Personal observation | Personal observation |
| AMNH FARB 1560 | *T. proriger* | Personal observation | Personal observation |
| AMNH FARB 1592 | *T. proriger* | Personal observation | Personal observation |
| FHSM VP-6907 | *T. proriger* | Personal observation | Personal observation |
| FHSM VP-2496 | *T. proriger* | Personal observation | Personal observation |
| KUVP 1033 | *T. proriger* | Personal observation | Personal observation |
| KUVP 50090 | *T. proriger* | Personal observation | Personal observation |
| KUVP 28705 | *T. proriger* | Personal observation | Personal observation |
| KUVP 65636 | *T. proriger* | Personal observation | Personal observation |
| KUVP 1020 | *T. proriger* | Personal observation | Personal observation |
| *AMNH FARB 1565 | *T. nepaeolicus* | Personal observation | Personal observation |
| AMNH FARB 124/134 | *T. nepaeolicus* | Everhart, 2002; Jiménez-Huidobro, Simões, & Caldwell, 2016; Russell, 1967; personal observation | Jiménez-Huidobro & Caldwell, 2019 and Russell, 1967; scale bar, measurements given, and personal observation |
| YPM 3980 | *T. nepaeolicus* | Everhart, 2002; Russell, 1967 | Russell, 1967; measurements given |
| YPM 3970 | *T. nepaeolicus* | Jiménez-Huidobro and Caldwell, 2019 | Jiménez-Huidobro and Caldwell, 2019 |
| YPM 3969 | *T. nepaeolicus* | Jiménez-Huidobro, Simões, & Caldwell, 2016 | Jiménez-Huidobro, Simões, & Caldwell, 2016; scale bar |
| YPM 3974 | *T. nepaeolicus* | Jiménez-Huidobro, Simões, & Caldwell, 2016 | Jiménez-Huidobro, Simões, & Caldwell, 2016 and Russell, 1967; scale bar and measurements given |
| AMNH FARB 1561 | *T. nepaeolicus* | Personal observation | Personal observation |
| FHSM VP-7262 | *T. nepaeolicus* | Personal observation | Personal observation |
| FHSM VP-2209 | *T. nepaeolicus* | Personal observation | Personal observation |
| YPM 3979 | *T. nepaeolicus* | Russell, 1967 | Russell, 1967; measurements given |
| YPM 3992 | *T. nepaeolicus* | Russell, 1967 | Russell, 1967; measurements given |
| YPM 4000 | *T. nepaeolicus* | Russell, 1967 | Russell, 1967; measurements given |
| YPM 3976 | *T. nepaeolicus* | Russell, 1967 | Russell, 1967; measurements given |
| AMNH FARB 2167 | *T. nepaeolicus* | Personal observation | Personal observation |
| *FHSM VP-2295 | *T. kansasensis* | Personal observation | Personal observation |
| FHSM VP-78 | *T. kansasensis* | Personal observation | Personal observation |
| FHSM VP-2495 | *T. kansasensis* | Personal observation | Personal observation |
| FHSM VP-3366 | *T. kansasensis* | Personal observation | Personal observation |
| FHSM VP-9350 | *T. kansasensis* | Personal observation | Personal observation |
| FHSM VP-13742 | *T. kansasensis* | Personal observation | Personal observation |
| FHSM VP-14848 | *T. kansasensis* | Personal observation | Personal observation |
| FHSM VP-15631 | *T. kansasensis* | Personal observation | Personal observation |
| FHSM VP-15632 | *T. kansasensis* | Personal observation | Personal observation |
| FGM V-43 | *T. kansasensis* | Personal observation | Personal observation |
| MCZ 1589 | *T. kansasensis* | Everhart, 2005 | Everhart, 2005; measurements and estimates given |
| YPM 40796 | *T. kansasensis* | Everhart, 2005 | Everhart, 2005; measurements and estimates given |
| LACMNH 127815 | *T. kansasensis* | Everhart, 2005 | Everhart, 2005; measurements and estimates given |
| TMM 40092-27 | *T. kansasensis* | Everhart, 2005 | Everhart, 2005; measurements given |
| TMM 81051-64 | *T. kansasensis* | Everhart, 2005 | Everhart, 2005; measurements given |
| IPB R322 | *T. kansasensis* | Jiménez-Huidobro, Simões, & Caldwell, 2016 | Jiménez-Huidobro, Simões, & Caldwell, 2016; scale bar |
| FHSM VP-17206 | *T. kansasensis* | Personal observation | Personal observation |
| FHSM VP-14840 | *T. kansasensis* | Personal observation | Personal observation |
| FMNH PR2103 | *T. kansasensis* | Personal observation | Personal observation |
| FMNH UC1342 | *T. kansasensis* | Personal observation | Personal observation |
| FHSM VP-18520 | *T. kansasensis* | Personal observation | Personal observation |
| FHSM VP-14841 | *Tylosaurus* sp. | Personal observation | Personal observation |
| FHSM VP-14842 | *Tylosaurus* sp. | Personal observation | Personal observation |
| FHSM VP-14843 | *Tylosaurus* sp. | Personal observation | Personal observation |
| FHSM VP-14844 | *Tylosaurus* sp. | Personal observation | Personal observation |
| FHSM VP-14845 | *Tylosaurus* sp. | Personal observation | Personal observation and estimates given by Konishi, Jiménez-Huidobro, & Caldwell, 2018 |

**References**

Cope ED. 1869. Remarks on *Macrosaurus proriger*. In Proceedings of the Academy of Natural

Sciences of Philadelphia, 11, 123.

Everhart MJ. 2002. New data on cranial measurements and body length of the mosasaur,

*Tylosaurus nepaeolicus* (Squamata; Mosasauridae), from the Niobrara Formation of

Western Kansas. Transactions of the Kansas Academy of Science 105:33-43.

Everhart MJ. 2005. *Tylosaurus* *kansasensis*, a new species of tylosaurine (Squamata,

Mosasauridae) from the Niobrara Chalk of western Kansas, USA. Netherlands Journal of

Geosciences 84:231-240.

Everhart MJ. 2017. Oceans of Kansas: a Natural History of the Western Interior Sea (second

edition). Indiana University Press, Bloomington, Indiana, 490 pp.

Everhart MJ. n.d. Oceans of Kansas Paleontology. www.oceansofkansas.com

Jiménez-Huidobro P, Simões TR, Caldwell MW. 2016. Re-characterization of *Tylosaurus*

*nepaeolicus* (Cope, 1874) and *Tylosaurus kansasensis* Everhart, 2005: ontogeny or

sympatry? Cretaceous Research 65:68-81.

Jiménez-Huidobro P, Caldwell MW. 2019. A new hypothesis of the phylogenetic relationships

of the Tylosaurinae (Squamata: Mosasauroidea). Frontiers in Earth Science 7:47.

Konishi T, Jiménez-Huidobro P, Caldwell MW. 2018. The smallest-known neonate individual of

*Tylosaurus* (Mosasauridae, Tylosaurinae) sheds new light on the Tylosaurine rostrum and

heterochrony. Journal of Vertebrate Paleontology 1-11.

Russell DA. 1967. Systematics and morphology of American mosasaurs. Yale University

Peabody Museum of Natural History Bulletin 23:1-241.

Stewart R F, Mallon J. 2018. Allometric growth in the skull of *Tylosaurus proriger* (Squamata:

Mosasauridae) and its taxonomic implications. Vertebrate Anatomy Morphology

Paleontology 6:75.
