# Supplementary material for: Craniofacial ontogeny in Tylosaurinae
Source: PeerJ. 2020 Oct 20;8:e10145. doi: 10.7717/peerj.10145 (PMC7583613; doi:10.7717/peerj.10145)
Supplement: Supplemental Information 9 — Single asterisks indicate specimens that were excluded from the analysis (i.e., wildcard specimens, specimens with incomplete or redundant coding) that produced the ontograms for each taxon, and double asterisks indicate specimens that were included in the single-taxon analyses but excluded from the test for anagenesis in Tylosaurus. KUVP 5033 was excluded from the analysis of growth in T. proriger but was included in the test of anagenesis; T. proriger YPM 3990 and T. proriger YPM 4002 were mentioned in the literature but could not be coded for any characters. [file peerj-08-10145-s009.docx]

| Specimen | Number of Characters Coded | % of Characters Coded |
| --- | --- | --- |
| *Tylosaurus* sp. |  |  |
| FHSM VP-14845 | 16 | 27.1 |
| *FHSM VP-14843 | 4 | 6.8 |
| *FHSM VP-14841 | 3 | 5.1 |
| *FHSM VP-14844 | 3 | 5.1 |
| *FHSM VP-14842 | 2 | 3.4 |
| *T. proriger* |  |  |
| AMNH FARB 4909 | 44 | 74.6 |
| FMNH P15144 | 43 | 72.9 |
| FHSM VP-3 | 38 | 64.4 |
| KUVP 1033 | 38 | 64.4 |
| KUVP 1032 | 35 | 59.3 |
| KUVP 66129 | 35 | 59.3 |
| RMM 5610 | 32 | 54.2 |
| *KUVP 65636 | 31 | 52.5 |
| FFHM 1997-10 | 31 | 52.5 |
| **CMN 8162 | 28 | 47.5 |
| AMNH FARB 221 | 28 | 47.5 |
| *KUVP 5033 | 25 | 42.4 |
| KUVP 28705 | 24 | 40.7 |
| **USNM 6086 | 22 | 37.3 |
| FMNH UR902 | 19 | 32.2 |
| *KUVP 1020 | 17 | 28.8 |
| **AMNH FARB 1555 | 15 | 25.4 |
| KUVP 50090 | 14 | 23.7 |
| CMN 51258-51263 | 12 | 20.3 |
| USNM 8898 | 12 | 20.3 |
| AMNH FARB 1592 | 11 | 18.6 |
| **TMP 1982.050.0010 | 10 | 16.9 |
| *AMNH FARB 1585 | 9 | 15.3 |
| **GSM 1 | 9 | 15.3 |
| ROM 7906 | 7 | 11.9 |
| **AMNH FARB 2160 | 6 | 10.2 |
| *MCZ 4374 | 4 | 6.8 |
| *KUVP 1129 | 4 | 6.8 |
| *FMNH UR820 | 4 | 6.8 |
| *FHSM VP-6907 | 4 | 6.8 |
| *HMG 1288 | 3 | 5.1 |
| *AMNH FARB 1543 | 2 | 3.4 |
| *YPM 3977 | 2 | 3.4 |
| *AMNH FARB 1560 | 2 | 3.4 |
| *YPM 1268 | 1 | 1.7 |
| *YPM 3981 | 1 | 1.7 |
| *FHSM VP-2496 | 1 | 1.7 |
| *YPM 3990 | 0 | 0 |
| *YPM 4002 | 0 | 0 |
| *T. nepaeolicus* |  |  |
| AMNH FARB 124/134 | 39 | 66.1 |
| FHSM VP-2209 | 35 | 59.3 |
| FHSM VP-7262 | 29 | 49.2 |
| YPM 3974 | 17 | 28.8 |
| AMNH FARB 1565 | 15 | 25.4 |
| **AMNH FARB 2167 | 13 | 22.0 |
| *AMNH FARB 1561 | 7 | 11.9 |
| **YPM 3970 | 6 | 10.2 |
| *YPM 3969 | 4 | 6.8 |
| *YPM 3992 | 2 | 3.4 |
| *YPM 4000 | 2 | 3.4 |
| *YPM 3980 | 1 | 1.7 |
| *YPM 3979 | 1 | 1.7 |
| *YPM 3976 | 1 | 1.7 |
| *T. kansasensis* |  |  |
| FHSM VP-2295 | 40 | 67.8 |
| FMNH PR2103 | 40 | 67.8 |
| FHSM VP-15632 | 38 | 64.4 |
| FHSM VP-78 | 33 | 55.9 |
| FGM V-43 | 31 | 52.5 |
| FHSM VP-9350 | 26 | 44.1 |
| **FHSM VP-3366 | 24 | 40.7 |
| FHSM VP-2495 | 18 | 30.5 |
| FHSM VP-15631 | 15 | 25.4 |
| *FHSM VP-13742 | 13 | 22.0 |
| **FHSM VP-18520 | 13 | 22.0 |
| *FHSM VP-14848 | 12 | 20.3 |
| *IPB R322 | 9 | 15.3 |
| **FHSM VP-17206 | 4 | 6.8 |
| *FMNH UC1342 | 4 | 6.8 |
| *FHSM VP-14840 | 3 | 5.1 |
| *MCZ 1589 | 2 | 3.4 |
| *LACMNH 127815 | 2 | 3.4 |
| *YPM 40796 | 1 | 1.7 |
| *TMM 40092-17 | 1 | 1.7 |
| *TMM 31051-64 | 1 | 1.7 |
