# Supplementary material for: Craniofacial ontogeny in Tylosaurinae
Source: PeerJ. 2020 Oct 20;8:e10145. doi: 10.7717/peerj.10145 (PMC7583613; doi:10.7717/peerj.10145)
Supplement: Supplemental Information 10 — Reversals are bold, phylogenetic characters are indicated by an asterisk, and characters that are purportedly diagnostic of T. proriger, T. kansasensis, or T. nepaeolicus are indicated by two asterisks. FHSM VP-14845 is a neonate referable to Tylosaurus sp. [file peerj-08-10145-s010.docx]

| Specimen | Growth Stage | Individual Variation |
| --- | --- | --- |
| Ontogram of *T. proriger* |  |  |
| FHSM VP-14845 | 1 | Quadrate suprastapedial process long** |
| AMNH FARB 2160 | 4 | Dorsal ridge of dentary predental process present |
| RMM 5610 | 5 | Parietal posterior pegs present* |
| KUVP 66129 | 6 | Distance between 1^st^ and 6^th^ maxillary teeth ≥ 25% TSL; parietal foramen touching or invading frontal-parietal suture**; frontal posterolateral processes robust; distance between 1^st^ and 6^th^ dentary teeth ≤ 35% dentary length |
| AMNH FARB 4909 | 9 | Premaxilla-maxilla suture rectangular; **quadrate tympanic ala deep****; parietal foramen touching or invading frontal-parietal suture** |
| KUVP 28705 | 9 | Premaxilla-maxilla suture u-shaped; parietal posterior pegs present* |
| KUVP 1033 | 9 | Dentary length between 60 and 56% lower jaw length |
| USNM 6086 | 10 | Dentary length between 60 and 56% lower jaw length |
| USNM 8898 | 10 | Distance between 1^st^ and 6^th^ maxillary teeth ≥ 25% TSL |
| FFHM 1997-10 | 11 | Distance between 1^st^ and 6^th^ maxillary teeth ≥ 25% TSL; parietal foramen touching or invading frontal-parietal suture** |
| KUVP 50090 | 12 | **Premaxillary rostrum length < 5% TSL** |
| KUVP 1032 | 13 | Frontal kite-shaped |
| GSM 1 | 14 | **TSL between 800–999 mm** |
| AMNH FARB 221 | 14 | **QH < 13% TSL** |
| FMNH P15144 | 15 | **Parietal nuchal fossa absent** |
| AMNH FARB 1555 | 16 | **Quadrate tympanic ala deep**** |
| FHSM VP-3 | 17 | **Quadrate suprastapedial process curved medially*** |
| Ontogram of *T. kansasensis/nepaeolicus* | |  |
| FHSM VP-2495 | 3 | Coronoid posteroventral process present |
| FHSM VP-78 | 4 | 12 dentary teeth* |
| FHSM VP-15632 | 5 | Quadrate suprastapedial process not curved medially*; **quadrate stapedial pit not defined**; parietal foramen touching or invading frontal-parietal suture** |
| FHSM VP-3366 | 6 | Distance between 1^st^ and 6^th^ dentary teeth < 35% dentary length |
| FHSM VP-2295 | 8 | Frontal medial suture flanges large**; parietal foramen touching or invading frontal-parietal suture** |
| AMNH FARB 1565 | 9 | Quadrate mandibular condyle anterodorsal deflection present |
| FMNH PR2103 | 10 | Premaxillary rostrum length ≥ 5% TSL; dorsal ridge of dentary predental process present; ≤ 12 dentary teeth* |
| FGM V-43 | 10 | Quadrate infrastapedial process rounded |
| AMNH FARB 2167 | 10 | QH 150–199 mm; **quadrate tympanic ala deep**** |
| AMNH FARB 124/134 | 11 | Quadrate suprastapedial process long**; **quadrate suprastapedial process slender** |
