# Supplementary material for: Craniofacial ontogeny in Tylosaurinae
Source: PeerJ. 2020 Oct 20;8:e10145. doi: 10.7717/peerj.10145 (PMC7583613; doi:10.7717/peerj.10145)
Supplement: Supplemental Information 11 — Reversals are bold, phylogenetic characters are indicated by an asterisk, and characters that are purportedly diagnostic of T. proriger, T. kansasensis, or T. nepaeolicus are indicated by two asterisks. [file peerj-08-10145-s011.docx]

| Specimen(s) | Growth Stage | Individual Variation |
| --- | --- | --- |
| FHSM VP-14845 (*T*sp.) | 1 | Quadrate suprastapedial process long** |
| FHSM VP-9350 (*Tk*) | 2 | Premaxillary rostrum distinctly knobbed, premaxilla-maxilla suture u-shaped, quadrate suprastapedial process thick, dentary length ≤ 55% lower jaw length |
| RMM 5610 (*Tp*) | 4 | Parietal posterior pegs present and small* |
| KUVP 66129 (*Tp*) | 5 | Distance between 1^st^ and 6^th^ maxillary teeth ≥ 25% TSL, 12 dentary teeth |
| AMNH FARB 4909 (*Tp*) | 6 | **Quadrate tympanic alar concavity deep****, **quadrate tympanic ala thin**** |
| KUVP 1033 (*Tp*) | 7 | TSL 800–999 mm, QH 100–149 mm |
| FGM V-43 (*Tk*), FHSM VP-2295 (*Tk*), FHSM VP-15632 (*Tk*), & FHSM VP-78 (*Tk*) | 8 | **Premaxillary rostrum < 5% TSL** |
| FGM V-43 (*Tk*) | 8 | TSL 800–999 mm, 12 maxillary teeth, quadrate suprastapedial process long**, **QH < 13% TSL** |
| FHSM VP-2295 (*Tk*), FHSM VP-15632 (*Tk*), & FHSM VP-78 (*Tk*) | 8 | **Quadrate infrastapedial process absent**** |
| FHSM VP-15632 (*Tk*) & FHSM VP-78 (*Tk*) | 8 | **TSL < 400 mm**, premaxillary rostrum foramina small**, **QH < 50 mm**, **quadrate mandibular condyle not completely ossified**, **dentary slender** |
| FHSM VP-15632 (*Tk*) | 8 | **Quadrate stapedial pit undefined** |
| FHSM VP-78 (*Tk*) | 8 | Quadrate suprastapedial process long**, **QH < 13% TSL**, **quadrate ala rim undefined**, **dentary dorsal ridge of predental process absent**, 12 dentary teeth* |
| FHSM VP-15631 (*Tk*) & FHSM VP-2495 (*Tk*) | 9 | **Jugal posteroventral angle nearly 90 degrees**** |
| FHSM VP-15631 (*Tk*) | 9 | **Coronoid posteroventral process absent** |
| FHSM VP-2495 (*Tk*) | 9 | **Dentary dorsal ridge of predental process absent** |
| YPM 3974 (*Tn*), AMNH FARB 124/134 (*Tn*), FHSM VP-2209 (*Tn*), FHSM VP-7262 (*Tn*), & FMNH PR2103 (*Tk*) | 10 | **Quadrate infrastapedial process absent****, parietal lateral borders straight**, 14 dentary teeth* |
| YPM 3974 (*Tn*) & AMNH FARB 124/134 (*Tn*) | 10 | Frontal dorsal midline crest absent**, **parietal nuchal fossa absent**, **distance between 1^st^ and 6^th^ dentary teeth > 35% dentary length** |
| AMNH FARB 124/134 | 10 | Quadrate suprastapedial process long** |
| FHSM VP-2209 (*Tn*), FHSM VP-7262 (*Tn*), & FMNH PR2103 (*Tk*) | 10 | **Dentary length 60–56% lower jaw length** |
| FHSM VP-2209 (*Tn*) | 10 | TSL 800–999 mm |
| FHSM VP-7262 (*Tn*) & FMNH PR2103 (*Tk*) | 10 | **Premaxillary rostrum without distinct knob**, **dentary dorsal ridge of predental process absent**, 12 dentary teeth* |
| FHSM VP-7262 (*Tk*) | 10 | Premaxillary rostrum foramina small, 12 maxillary teeth*, **parietal nuchal fossa absent**, **jugal posteroventral process absent***, **dentary slender** |
| KUVP 1032 (*Tp*), KUVP 50090 (*Tp*), USNM 8898 (*Tp*), FFHM 1997-10 (*Tp*), FMNH P15144 (*Tp*), ROM 7906 (*Tp*), & AMNH FARB 221 (*Tp*) | 11 | Premaxilla-maxilla suture m-shaped |
| KUVP 1032 (*Tp*), KUVP 50090 (*Tp*), USNM 8898 (*Tp*), & FFHM 1997-10 (*Tp*) | 11 | **Distance between 1^st^ and 6^th^ dentary teeth > 35% dentary length** |
| KUVP 1032 (*Tp*) | 11 | Frontal kite-shaped |
| KUVP 50090 (*Tp*), USNM 8898 (*Tp*), & FFHM 1997-10 (*Tp*) | 11 | **Distance between 1^st^ and 6^th^ dentary teeth > 25% TSL** |
| KUVP 50090 (*Tp*) | 11 | **Premaxillary rostrum < 5% TSL** |
| USNM 8898 (*Tp*) & FFHM 1997-10 (*Tp*) | 11 | **Premaxillary rostrum without distinct knob**, distance between 1^st^ and 6^th^ maxillary teeth ≥ 25% TSL |
| USNM 8898 (*Tp*) | 11 | **TSL 400–800 mm**, **dentary length > 60% lower jaw length** |
| FMNH P15144 (*Tp*), ROM 7906 (*Tp*), & AMNH FARB 221 (*Tp*) | 11 | **Dentary slender** |
| FMNH P15144 (*Tp*) | 11 | **Dentary length 60–56% lower jaw length**, coronoid posteroventral process present and fan-like |
| ROM 7906 (*Tp*) & AMNH FARB 221 (*Tp*) | 11 | **QH 100–150 mm** |
| AMNH FARB 221 (*Tp*) | 11 | **QH < 13% TSL** |
| FHSM VP-3 (*Tp*) | 12 | **Dentary length 60–56% lower jaw length** |
| KUVP 5033 (*Tp*) | 13 | TSL ≥ 1400 mm, QH ≥ 200 mm |
