# Supplementary material for: Craniofacial ontogeny in Tylosaurinae
Source: PeerJ. 2020 Oct 20;8:e10145. doi: 10.7717/peerj.10145 (PMC7583613; doi:10.7717/peerj.10145)
Supplement: Supplemental Information 12 — Asterisks indicate estimates from the literature. [file peerj-08-10145-s012.docx]

| Specimen | Growth Rank | TSL (mm) | TSL Size Rank | QH (mm) | QH Size Rank |
| --- | --- | --- | --- | --- | --- |
| FHSM VP-14845 | 1 | 300* | 1 | 30* | 1 |
| CMN 51258-51263 | 2 | - | - | 70* | 3 |
| AMNH FARB 1592 | 3 | - | - | 71 | 4.5 |
| FMNH UR902 | 4 | - | - | 75 | 7 |
| RMM 5610 | 6 | 611* | 6 | 72* | 6 |
| KUVP 66129 | 7 | 506 | 2 | 63 | 2 |
| CMN 8162 | 8 | 574 | 3 | 71 | 4.5 |
| AMNH FARB 4909 | 10.5 | 610 | 5 | 78 | 8 |
| TMP 1982.050.0010 | 10.5 | 810 | 9 | 111 | 11 |
| KUVP 1033 | 10.5 | 813 | 10 | 106 | 10 |
| KUVP 28705 | 10.5 | 615 | 7 | - | - |
| USNM 6086 | 13.5 | 585 | 4 | 79 | 9 |
| USNM 8898 | 13.5 | 710 | 8 | - | - |
| FFHM 1997-10 | 15 | 1016 | 13 | 150 | 15 |
| KUVP 50090 | 16 | 1300 | 18 | - | - |
| KUVP 1032 | 17 | 1212 | 17 | 170 | 18 |
| AMNH FARB 221 | 19 | 1180 | 15 | 135 | 13 |
| GSM 1 | 19 | 980 | 11 | 133 | 12 |
| ROM 7906 | 19 | 1005 | 12 | 144 | 14 |
| FMNH P15144 | 21 | 1201 | 16 | 173 | 19 |
| AMNH FARB 1555 | 22 | - | - | 151 | 16 |
| FHSM VP-3 | 23 | 1130 | 14 | 165 | 17 |
