# Supplementary material for: Craniofacial ontogeny in Tylosaurinae
Source: PeerJ. 2020 Oct 20;8:e10145. doi: 10.7717/peerj.10145 (PMC7583613; doi:10.7717/peerj.10145)
Supplement: Supplemental Information 13 — Single asterisks indicate estimates by the author, and double asterisks are estimates from the literature. [file peerj-08-10145-s013.docx]

| Specimen | Growth Rank | TSL (mm) | TSL Size Rank | QH (mm) | QH Size Rank |
| --- | --- | --- | --- | --- | --- |
| FHSM VP-14845 | 1 | 300* | 1 | 30* | 1 |
| FHSM VP-9350 | 3.5 | - | - | 37 | 2 |
| FHSM VP-78 | 5 | 378 | 5 | 43 | 3 |
| FHSM VP-15632 | 6 | 360** | 6 | 46 | 4 |
| FHSM VP-7262 | 8 | - | - | 106 | 12 |
| FHSM VP-3366 | 8 | - | - | 93 | 11 |
| FHSM VP-2295 | 11 | 650 | 11 | 82 | 6.5 |
| AMNH FARB 1565 | 12 | - | - | 78 | 5 |
| AMNH FARB 2167 | 14 | - | - | 155 | 15 |
| FGM V-43 | 14 | 890** | 14 | 88 | 9 |
| FMNH PR2103 | 14 | 653 | 14 | 87 | 8 |
| YPM 3974 | 16.5 | - | - | 82* | 6.5 |
| AMNH FARB 124/134 | 16.5 | 717 | 16.5 | 92 | 10 |
| YPM 3970 | 17.5 | - | - | 121 | 13 |
| FHSM VP-2209 | 17.5 | 851** | 11 | 133 | 14 |
