# Supplementary material for: Craniofacial ontogeny in Tylosaurinae
Source: PeerJ. 2020 Oct 20;8:e10145. doi: 10.7717/peerj.10145 (PMC7583613; doi:10.7717/peerj.10145)
Supplement: Supplemental Information 14 — Single asterisks indicate estimates by the author, and double asterisks are estimates from the literature. [file peerj-08-10145-s014.docx]

| Specimen | Growth Rank | TSL (mm) | TSL Size Rank | QH (mm) | QH Size Rank |
| --- | --- | --- | --- | --- | --- |
| FHSM VP-14845 (*T*sp.) | 1 | 300* | 1 | 30* | 1 |
| FHSM VP-9350 (*Tk*) | 2 | - | - | 37 | 2 |
| AMNH FARB 1592 (*Tp*) | 3.5 | - | - | 71 | 6 |
| FMNH UR902 (*Tp*) | 3.5 | - | - | 75 | 8 |
| RMM 5610 (*Tp*) | 5.5 | 611* | 6 | 72* | 7 |
| AMNH FARB 1565 (*Tn*) | 5.5 | - | - | 78 | 9.5 |
| KUVP 66129 (*Tp*) | 7 | 506 | 4 | 63 | 5 |
| AMNH FARB 4909 (*Tp*) | 8 | 610 | 5 | 78 | 9.5 |
| KUVP 1033 (*Tp*) | 9 | 813 | 13 | 106 | 16.5 |
| KUVP 28705 (*Tp*) | 12 | 615 | 7 | - | - |
| FHSM VP-78 (*Tk*) | 12 | 378 | 3 | 43 | 3 |
| FHSM VP-15632 (*Tk*) | 12 | 360** | 2 | 46 | 4 |
| FHSM VP-2295 (*Tk*) | 12 | 650 | 8 | 82 | 11.5 |
| FGM V-43 (*Tk*) | 12 | 890** | 15 | 88 | 14 |
| FHSM VP-7262 (*Tn*) | 19 | - | - | 106 | 16.5 |
| FMNH PR2103 (*Tk*) | 19 | 653 | 9 | 87 | 13 |
| YPM 3974 (*Tn*) | 19 | - | - | 82* | 11.5 |
| AMNH FARB 124/134 (*Tn*) | 19 | 717 | 12 | 92 | 15 |
| FHSM VP-2209 (*Tn*) | 19 | 851** | 14 | 133 | 18 |
| USNM 8898 (*Tp*) | 25 | 710 | 10 | - | - |
| ROM 7906 (*Tp*) | 25 | 1005 | 16 | 144 | 20 |
| FFHM 1997-10 (*Tp*) | 25 | 1016 | 17 | 150 | 22 |
| AMNH FARB 221 (*Tp*) | 25 | 1180 | 19 | 135 | 19 |
| FMNH P15144 (*Tp*) | 25 | 1201 | 20 | 173 | 25 |
| KUVP 1032 (*Tp*) | 25 | 1212 | 21 | 170 | 24 |
| KUVP 50090 (*Tp*) | 25 | 1300 | 22 | - | - |
| FHSM VP-3 (*Tp*) | 29 | 1130 | 18 | 165 | 23 |
| KUVP 5033 (*Tp*) | 30 | 1700** | 23 | 225 | 26 |
